# Fully automated and high-fidelity robotic platform enabling accelerated discovery of nanocatalysts

**DOI:** 10.1039/d5sc06192j

**Published:** 2025-12-30

**Authors:** Shin Wook Kang, Kyung Hee Oh, Kanghoon Yim, Sanha Jang, Jin Gyu Lee, Jung-Il Yang, Ji Chan Park

**Affiliations:** a Clean Fuel Research Laboratory, Korea Institute of Energy Research Daejeon 34129 Republic of Korea jcpark@kier.re.kr; b Energy AI & Computational Science Laboratory, Korea Institute of Energy Research Daejeon 34129 Republic of Korea; c Energy Engineering, University of Science and Technology Daejeon 34113 Republic of Korea

## Abstract

The discovery of heterogeneous catalysts increasingly relies on high-throughput experimentation and high-fidelity data. Here, we report a fully automated robotic platform that integrates two synchronized collaborative robotic arms, automated liquid handling, and time-resolved UV-Vis kinetic analysis for the rapid, reproducible, and data-rich evaluation of nanocatalysts. Unlike existing high-throughput systems, which often compromise data quality, our platform combines parallel reaction execution with real-time processing of time-resolved measurements and automated performance ranking, thereby delivering both speed and precision. Using the catalytic reduction of 4-nitrophenol as a benchmark, we screened 24 Pd-based catalysts including 22 metal-added Pd/AC variants, and completed 96 measurements in 16 h 40 min achieving an average throughput of ∼10 min per sample. The system achieved high reproducibility, with relative standard deviations of approximately 2%, and detected subtle kinetic differences such as the enhanced activity of catalysts containing Fe, Cu, Zn, and Sn. Correlating experimental performance with density functional theory (DFT)-derived descriptors revealed structure–activity relationships and highlighted nanoscale effects not captured by bulk calculations.

## Abstract 2

A fully automated robotic platform integrates sample preparation, time-resolved UV-Vis monitoring, and real-time data processing for high-throughput, high-fidelity screening of Pd-based nanocatalysts in 4-nitrophenol reduction.

## Graphical abstract

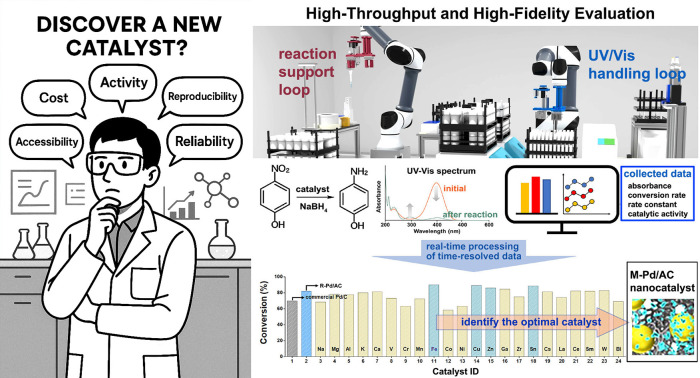

## Introduction

Catalysts are essential to modern chemical industries, participating in over 90% of all chemical manufacturing processes and playing critical roles in the advancement of energy technologies, materials development, and pharmaceuticals.^1–3^ The performance of catalytic reactions, typically characterized by activity, selectivity, and stability, depends heavily on the discovery and optimization of advanced catalytic materials.^4–9^ However, conventional methods for catalyst development remain largely manual, labor-intensive, and low throughput.^10–14^ These limitations continue to hinder progress in heterogeneous catalysis, where the demand for efficient, reproducible, and scalable methodologies is rapidly increasing.

To accelerate the discovery process, high-throughput experimentation (HTE) and computational modeling have emerged as powerful, complementary strategies.^15–19^ Atomistic simulations assist in identifying promising catalyst candidates and narrowing the experimental search space.^20–24^ Nonetheless, computational methods often fail to capture the full complexity of real-world systems, where phenomena such as mass transport limitations, catalyst restructuring, and surface impurities can strongly affect reactivity. Thus, physical experimentation remains indispensable for validating theoretical predictions and uncovering emergent catalytic behaviors. In this context, laboratory automation, particularly the integration of robotics with time-resolved and fully automated analytical workflows has gained growing attention for enhancing reproducibility, reducing human error, and increasing throughput.^25–27^

Recent advances in automated synthesis, parallel reactor arrays, and microfluidic screening systems have demonstrated the feasibility of automation in catalyst discovery.^28–32^ While high-throughput systems often maximize experimental efficiency, maintaining consistent analytical fidelity across many samples remains a challenge. For example, differences in reactor geometry, mixing strategy, or handling protocols can directly impact kinetic reproducibility.^33^ A recent review also highlights the importance of balancing throughput with data integrity in automated workflows.^34^ Inconsistencies in sample handling, non-standardized analytical protocols, and fragmented data workflows frequently result in limited reproducibility.^35,36^ These issues hinder the adoption of such systems in data-driven methodologies, including machine learning. Moreover, the absence of integrated automated kinetic-analysis modules limits mechanistic interpretation and hinders accurate benchmarking, both of which are critical for rational catalyst design.^37,38^

Model reactions are essential for evaluating the reliability of automated catalyst screening platforms. Among these, the catalytic reduction of 4-nitrophenol (4-NP) has been widely used due to its simple and well-defined reaction mechanism, clear UV-Vis spectroscopic signal, and environmental relevance.^39–41^ As an industrial pollutant, 4-NP is a key target for remediation, and its reduction product, 4-aminophenol (4-AP), is an important intermediate in the manufacture of pharmaceuticals and dyes. The reaction's straightforward kinetics and quantifiable conversion make it a suitable benchmark for automated, high-throughput catalyst evaluation.

In this study, we report a fully automated robotic platform for high-throughput and high-fidelity evaluation of Pd-based nanocatalysts. The system integrates two collaborative robotic arms, automated liquid handling, and in-line UV-Vis spectroscopy to enable seamless catalyst testing with minimal human intervention. A custom software suite performs real-time processing of time-resolved kinetic measurements and automated performance ranking. Although our platform adopts the efficiency-oriented philosophy of combinatorial chemistry, it is fundamentally different in its level of automation and its integration of quantitative, time-resolved kinetic analysis. Instead of qualitative or single-point screening, the system performs fully automated kinetic measurements that yield high-fidelity datasets well suited for machine-learning-driven analysis and autonomous optimization. These capabilities establish the platform as a robust foundation for next-generation, AI-integrated catalyst discovery.

To demonstrate the performance and reliability of the automated workflow, this 24-member catalyst library was intentionally selected as a proof-of-concept set to validate throughput, reproducibility, and workflow stability before scaling to larger compositional spaces. Although the present study focuses on a simplified model reaction, the modular design of the platform readily supports future expansion to substantially broader catalyst libraries and more complex catalytic transformations.

Using the 4-NP reduction as a model reaction, we screened 24 Pd-based catalysts, comprising 22 metal-added Pd catalysts supported on activated charcoal (M-Pd/AC, where M = Fe, Cu, Zn, *etc.*), a reference Pd/AC catalyst (R-Pd/AC), and a commercial Pd/C catalyst. The platform successfully processed 96 reactions in a single overnight run, and its automated handling and standardized measurement workflow enabled the generation of highly reproducible, time-resolved kinetic datasets. These high-quality data also enabled direct comparison of experimental performance with computational predictions. By correlating screening results with density functional theory (DFT)-derived descriptors such as alloy formation energy, d-band center, and work function from the Materials Project database, we evaluate how well bulk-scale properties reflect actual nanoscale catalytic behavior and identify cases where theory and experiment converge or diverge.

## Results and discussion

### Design of robotic platform and automated workflow

**Fig. 1:**
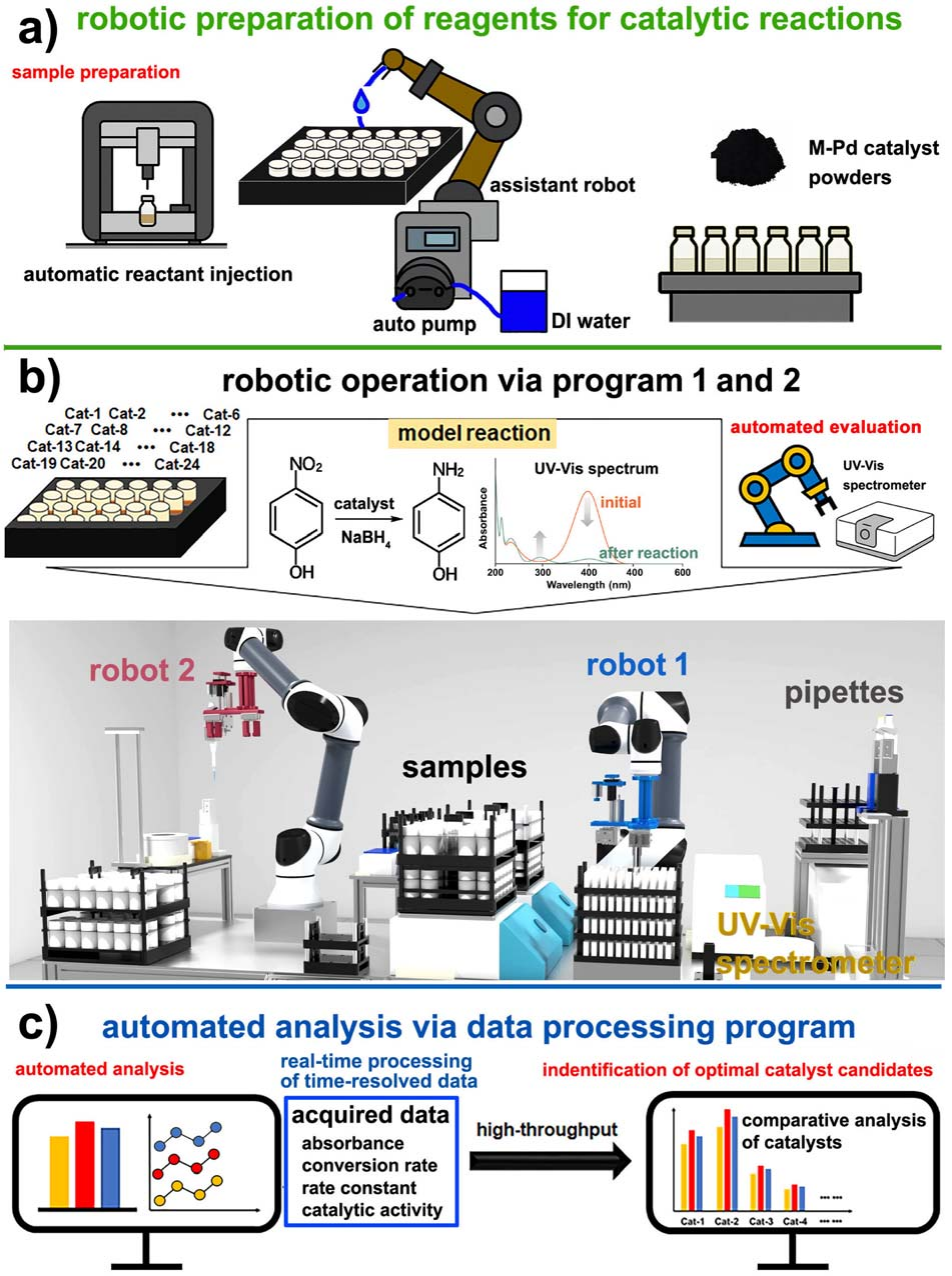
Conceptual design and workflow of the automated robotic platform. (a) Sample preparation *via* robotic liquid handling and auto-dispensing modules for catalyst slurries and reagents. (b) Robotic execution of the model reaction (4-NP reduction) with time-resolved UV-Vis monitoring based on discrete aliquot sampling. (c) Conceptual diagram illustrating data analysis and catalyst activity ranking performed through custom software that integrates real-time processing of time-resolved measurements with automated kinetic evaluation and visualization.

To enable high-throughput and reproducible catalyst screening, we designed an automated robotic platform that integrates sample preparation, reaction execution, and kinetic analysis into a unified workflow (Fig. 1). A conceptual 3D animation illustrating the overall system architecture and the operation of each robotic module is provided for reference (Movie S1 in the SI). The system is composed of three functional modules: (i) robotic sample preparation, (ii) catalytic reaction execution with automated data acquisition, and (iii) real-time processing of time-resolved data. In the sample preparation stage, M-Pd/AC catalyst powders are preloaded into reaction vessels. A robotic arm, coupled with an auto-pump system, dispenses deionized water into each sample bottle, while the reactant solution is delivered by the automated liquid handling system. This separation of tasks minimizes operator-induced variability and ensures consistency across all test samples (Fig. 1a). In the reaction execution stage, NaBH_4_ solution is added to initiate the reduction of 4-NP to 4-AP, a widely used model reaction for benchmarking catalytic activity. Two collaborative robotic arms coordinate liquid handling and in-line UV-Vis spectroscopic measurements. The reaction progress is monitored through discrete, time-resolved sampling by measuring the absorbance at 400 nm at fixed intervals (Fig. 1b). All kinetic data, including absorbance profiles, conversion percentages, rate constants, and activity metrics, are automatically processed using custom-built software. The analysis includes pseudo-first-order kinetic fitting based on ln(*A*_t_/*A*_0_) *versus* time plots and produces key visualizations such as kinetic traces, bar graphs, and real-time comparisons across catalysts (Fig. 1c).

**Fig. 2:**
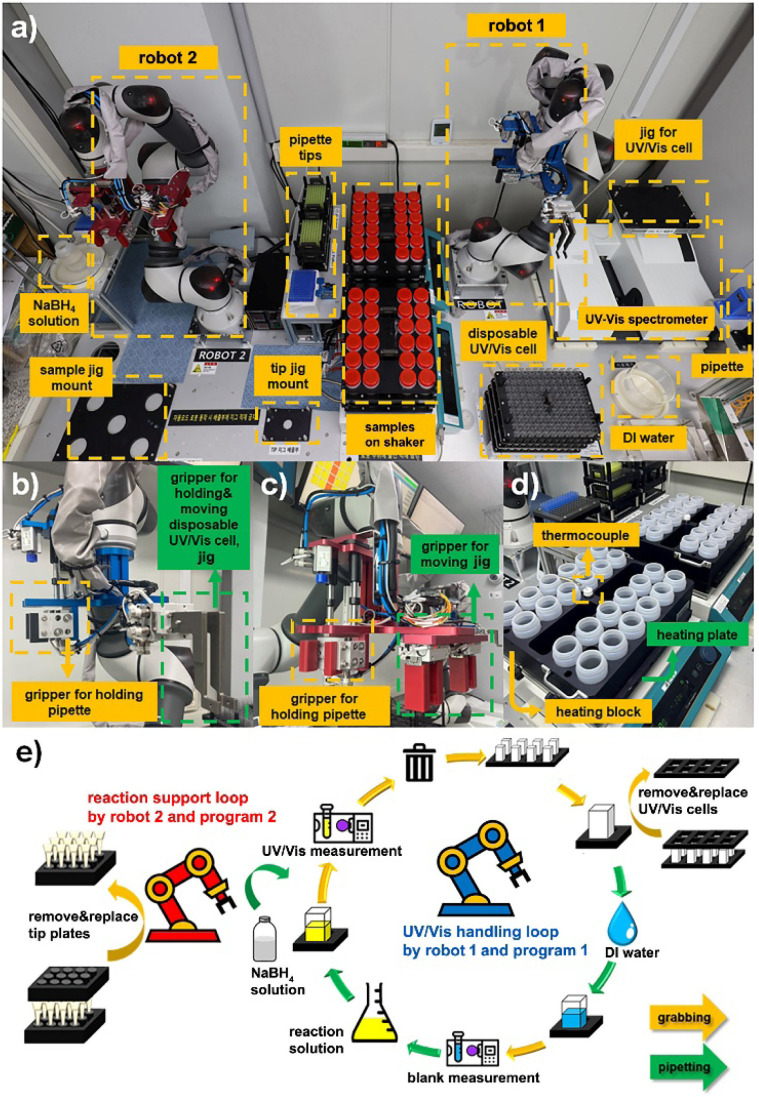
Integrated robotic system architecture and workflow for high-throughput catalyst screening. (a) Overhead view of the platform layout showing robot 1 (right), responsible for pipetting and UV-Vis measurements, and robot 2 (left), responsible for NaBH_4_ injection and jig management. Key modules include sample jigs, shaker, tip mounts, UV-Vis spectrometer, and DI water supply. (b) Gripper configuration of robot 1 for handling pipettes and disposable UV-Vis cells. (c) Gripper configuration of robot 2 for holding pipettes and managing jig movements. (d) Heating plate and block assembly with integrated thermocouples for temperature-controlled reactions. (e) Schematic diagram of the automated dual-loop operation: robot 1 and program 1 manage the UV-Vis loop (blank scan, dilution, measurement), while robot 2 and program 2 execute the reaction loop (NaBH_4_ injection, waste handling).

The platform provides a physical implementation of the integration of hardware components designed to realize the conceptual workflow (Fig. 2 and S1). Each robotic subsystem was strategically designed to operate with minimal user intervention while maximizing throughput and fidelity. The overall platform layout consists of two collaborative robotic arms placed on opposite sides of a modular workbench (Fig. 2a). Robot 1 is responsible for UV-Vis sampling and measurement, while robot 2 supports the reaction setup by handling pipette tips, delivering NaBH_4_ solution, and managing the heating/shaking jigs.

Each reagent station that involves NaBH_4_, deionized (DI) water, catalyst bottles, and disposable UV-Vis cells, is preloaded and aligned to the robots' path planning, ensuring uninterrupted sequential operation. These interfaces improve reproducibility by preventing user-input errors, enforcing predefined process sequences, and providing visual tracking of pipette tips and UV-Vis cell usage. They also minimize human intervention and streamline high-throughput catalyst screening. The robot control program 1 includes an integrated execution dashboard that enables users to configure process timing, robotic operations, and experimental sequences (Fig. S2). Visual management tools for UV-Vis cells and pipette tips ensure proper resource tracking and prevent operational errors (Fig. S3 and S4). The shaker configuration panel enables precise control of sample mixing conditions (Fig. S5). These interfaces enhance reproducibility, minimize human intervention, and streamline high-throughput catalyst screening.

To accommodate diverse manipulation tasks, two robotic arms are equipped with custom-designed multi-tool grippers. Robot 1 alternates between pipette tools and UV-Vis cell holders, enabling precise movements that ensure each UV-Vis measurement is conducted under standardized conditions, thereby minimizing variability in spectral data collection (Fig. 2b). The disposable pipette tip and UV-Vis cell system is managed through automated replacement, minimizing optical contamination and measurement noise. Robot 2 is equipped with specialized grippers to handle pipettes and reposition entire sample trays, enabling efficient dispensing of NaBH_4_ precisely synchronized after the baseline UV-Vis scan to ensure uniform reaction start times (Fig. 2c). The tip-tracking interface monitors the status of reaction pipette tips and coordinates sequential processes during reagent mixing and jig reclamation to ensure seamless automation by robot control program 2 (Fig. S6). The system's ability to handle 96 reactions in parallel without human intervention strongly enhances experimental consistency and temporal control. Temperature regulation and reaction homogeneity are achieved through the use of custom-designed heating blocks and integrated thermocouples (Fig. 2d). The thermally conductive blocks maintain the desired reaction temperature across all bottles, while orbital shaking ensures uniform dispersion of reactants and catalysts. Thermal feedback loops, recorded by embedded thermocouples, provide real-time validation of the reaction environment and further support kinetic reproducibility. The system-wide automation protocol is schematically summarized, with robotic operations divided into two interdependent loops: the UV-Vis handling loop managed by Robot 1 and the reaction support loop managed by Robot 2 (Fig. 2e and Movie S2). Programmed in a modular and parallelized manner, these two operational loops allow each robot to function continuously without mutual interference. Robot 1 is responsible for dilution, UV-Vis cuvette handling, and spectroscopic analysis, whereas Robot 2 performs reagent addition for reaction initiation, pipette tip replacement, and sample tray resetting. This workflow architecture enables the complete screening of a 96-catalyst library within a single day (Movie S3).

Moreover, the integration of synchronized robotic actions with automated kinetic analysis software allows for real-time generation of catalytic performance metrics, including conversion, rate constant, and active metal activity (Fig. S7). This streamlined operation not only reduces labor and analysis time, but also minimizes error propagation from manual pipetting, reaction timing discrepancies, or UV-Vis cell misalignment. The platform's high correlation coefficients across replicate trials attest to its robust reproducibility and precision. Collectively, the platform offers a powerful solution for scalable catalyst discovery and optimization, capable of handling combinatorial libraries with minimal human supervision.

### Catalyst synthesis and sample uniformity

**Fig. 3:**
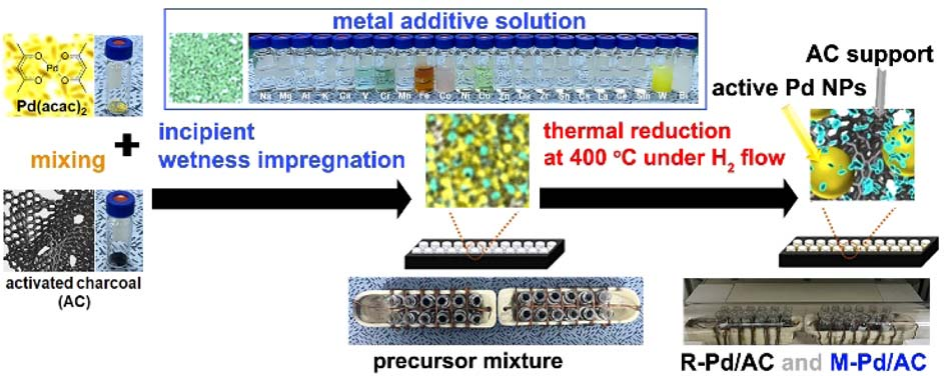
Schematic illustration of the synthesis workflow for R-Pd/AC and M-Pd/AC nanocatalysts. The procedure involves wet impregnation of Pd(acac)_2_ and activated charcoal, addition of metal additive precursors (10 mol% relative to Pd) for M-Pd/AC variants, drying, and hydrogen reduction at 400 °C to Pd nanoparticles.

The catalyst synthesis protocol, illustrated schematically, demonstrates a wet-impregnation-based, one-pot preparation method that enables the simultaneous and consistent synthesis of a large number of catalysts, including both reference (R-Pd/AC) and metal-added (M-Pd/AC) variants (Fig. 3). The procedure begins with homogeneous mixing of Pd(acac)_2_ and activated charcoal (AC), followed by the precise addition of metal additive precursors, introducing compositional diversity across 22 metal variants within the library (Table S1). To achieve fine control despite the small volumes involved, additive precursors are first dissolved in ethanol and added *via* stock solutions, allowing accurate dosing at 10 mol% relative to Pd. This modular synthetic strategy facilitates compatibility with automated and scalable preparation of multiple catalyst samples in parallel.

Importantly, the selection of metal additives was also driven by economic considerations: rather than using costly noble-metal promoters such as Pt, Ir, or Rh, we employed low-cost, earth-abundant first-row transition-metal salts (*e.g.*, Fe, Co, Ni, Cu). Despite their minimal cost, these additives noticeably modulated and in some cases improved Pd catalytic performance, effectively reducing the cost per unit activity and providing a practical, economically favorable route to catalyst optimization.

Once prepared, the precursor slurries are dried and subjected to thermal reduction at 400 °C under flowing H_2_, a condition optimized to promote nanoparticle formation while minimizing aggregation. The process reproducibly yields finely dispersed Pd nanoparticles, with or without the added metal species, across a full 24-sample batch. All catalyst samples used in the robotic screening platform were synthesized using this unified protocol, ensuring that observed performance differences arise solely from compositional variations rather than procedural inconsistencies.

### Performance benchmarking and kinetic evaluation

To benchmark the reliability and resolution of the robotic screening system, we conducted a side-by-side comparison of R-Pd/AC (synthesized *via* wet-impregnation) and commercial Pd/C under identical conditions for the NaBH_4_-mediated reduction of 4-NP. To ensure consistency and minimize human error, a dedicated data processing program was developed to automate key calculations, including conversion, rate constant, and catalytic activity metrics. The software automatically extracts absorbance values at 400 nm and applies a pseudo-first-order kinetic model to derive relevant parameters.

Accurate execution of these calculations requires prior input of catalyst information, including metal content, catalyst mass, and reactant molar amount (Fig. S8). The experimental parameters for each dataset are summarized to enable transparent and reproducible data analysis based on real-time monitoring (Fig. S9).

**Fig. 4:**
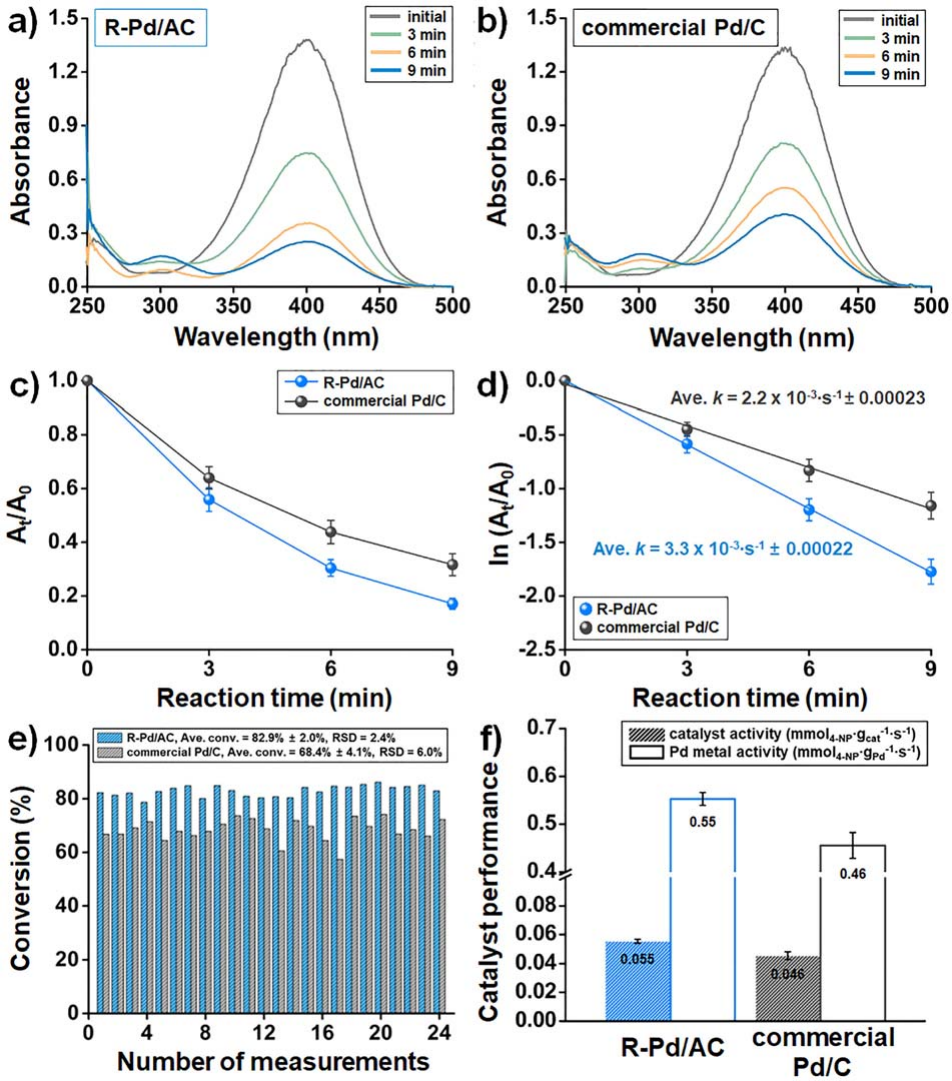
Benchmarking of catalytic performance between R-Pd/AC and commercial Pd/C at 25 °C across 24 replicate measurements. (a and b) Representative UV-Vis absorbance spectra measured at 3 min intervals during 4-NP reduction for (a) R-Pd/AC and (b) commercial Pd/C. (c) Reaction progress represented by *A*_t_/*A*_0_ as a function of time. (d) Pseudo-first-order kinetic fits from ln(*A*_t_/*A*_0_) *versus* time, yielding rate constants (*k*) and standard deviations for both catalysts. (e) Comparison of 4-NP conversion after 9 min. (f) Summary of catalytic performance expressed as catalyst activity and Pd metal activity, highlighting the higher activity and reproducibility of R-Pd/AC.

UV-Vis spectra recorded at 3 min intervals show that R-Pd/AC exhibited a more rapid decrease in the 400 nm peak, indicating faster reaction kinetics (Fig. 4a and b). A 3 min interval was selected as a practical compromise: although 1 min measurements are technically feasible, the large number of samples would place excessive demand on the UV-Vis instrument. Furthermore, 3 min intervals provided sufficient temporal resolution to effectively capture the reaction progression. Time-resolved absorbance profiles (*A*_t_/*A*_0_) clearly demonstrate that R-Pd/AC facilitates more efficient reduction of 4-NP over time compared to commercial Pd/C (Fig. 4c). The greater slope in the R-Pd/AC curve indicates higher catalytic activity, which is further supported by kinetic fitting. Using the pseudo-first-order rate model, ln(*A*_t_/*A*_0_) *vs.* time yielded a rate constant (*k*) of 3.3 × 10^−3^ s^−1^ ± 0.00022 for R-Pd/AC and 2.1 × 10^−3^ s^−1^ ± 0.00023 for commercial Pd/C (Fig. 4d). The conversion efficiency was calculated using conversion (%) = (1 − *A*_t_/*A*_0_) *×* 100, at 9 min across 24 replicates. R-Pd/AC achieved a mean conversion of 82.9% ± 2.0%, which was significantly higher and more consistent than that of commercial Pd/C (68.4% ± 4.1%) (Fig. 4e). The lower relative standard deviation (RSD) for R-Pd/AC (2.4%) compared to commercial Pd/C (6.0%) confirms better reproducibility.

Catalytic performance metrics, including catalyst activity normalized by total catalyst mass and Pd metal activity normalized by Pd mass, are summarized to highlight relative efficiency (Fig. 4f). The R-Pd/AC catalyst demonstrated superior catalytic performance, with a catalytic activity of 0.055 mmol_4-NP_ g_cat_^−1^ s^−1^ ± 0.001 and a Pd metal activity of 0.55 mmol_4-NP_ g_Pd_^−1^ s^−1^ ± 0.01, both ∼1.2-fold higher than those of commercial Pd/C (0.046 mmol_4-NP_ g_cat_^−1^ s^−1^ ± 0.003 and 0.46 mmol_4-NP_ g_Pd_^−1^ s^−1^ ± 0.03). These results collectively underscore the superior catalytic performance of R-Pd/AC, not only in terms of activity but also in operational consistency. The steeper decline in 4-NP absorbance and the higher rate constant further confirm the improved kinetic performance of R-Pd/AC, which can be attributed to more uniform metal dispersion and optimized nanoparticle formation.

To further assess the precision and reproducibility of the developed robotic system, we benchmarked its performance against a conventional manual workflow using the same commercial Pd/C catalyst. In the manual procedure, all experimental steps including catalyst weighing, reagent addition, and sampling for UV-Vis measurement were conducted by hand using stirrer, micropipettes, a stopwatch, and a benchtop UV-Vis spectrometer (Fig. S10a and b). Both the automated and manual workflows were evaluated using 24 independent replicate reactions (*n* = 24), each conducted in separate reaction vessels prepared under identical conditions. Despite careful manual execution, the catalytic reduction experiments exhibited noticeable variability, yielding an average conversion of 75.9% ± 6.7%, with an RSD of 8.8% across 24 trials (Fig. S10c and d). In contrast, the robotic platform produced a slightly lower average conversion of 68.4% ± 4.1%, but achieved markedly better reproducibility, reflected in a lower RSD of 6.0%. This comparison underscores the superior precision and consistency of the automated system, which minimizes human-induced variation and ensures reliable catalyst performance evaluation. The observed variability in manual runs likely stems from inconsistencies in pipetting, reaction timing, and solution handling, all of which are mitigated through automation.

These results underscore the key benefits of robotic automation for catalyst screening: minimized operator-induced errors, improved experimental repeatability, and enhanced data quality. Beyond the initial setup, the platform performs all experimental steps autonomously, increasing practical throughput from ∼3 manually processed samples per day to nearly 100 automated measurements per day. This substantial reduction in human labor further demonstrates the efficiency and long-term cost-effectiveness of the automated workflow. Furthermore, the system's ability to discern subtle kinetic differences under identical conditions validates its utility not only for high-throughput screening but also for benchmarking and mechanistic investigations.

**Fig. 5:**
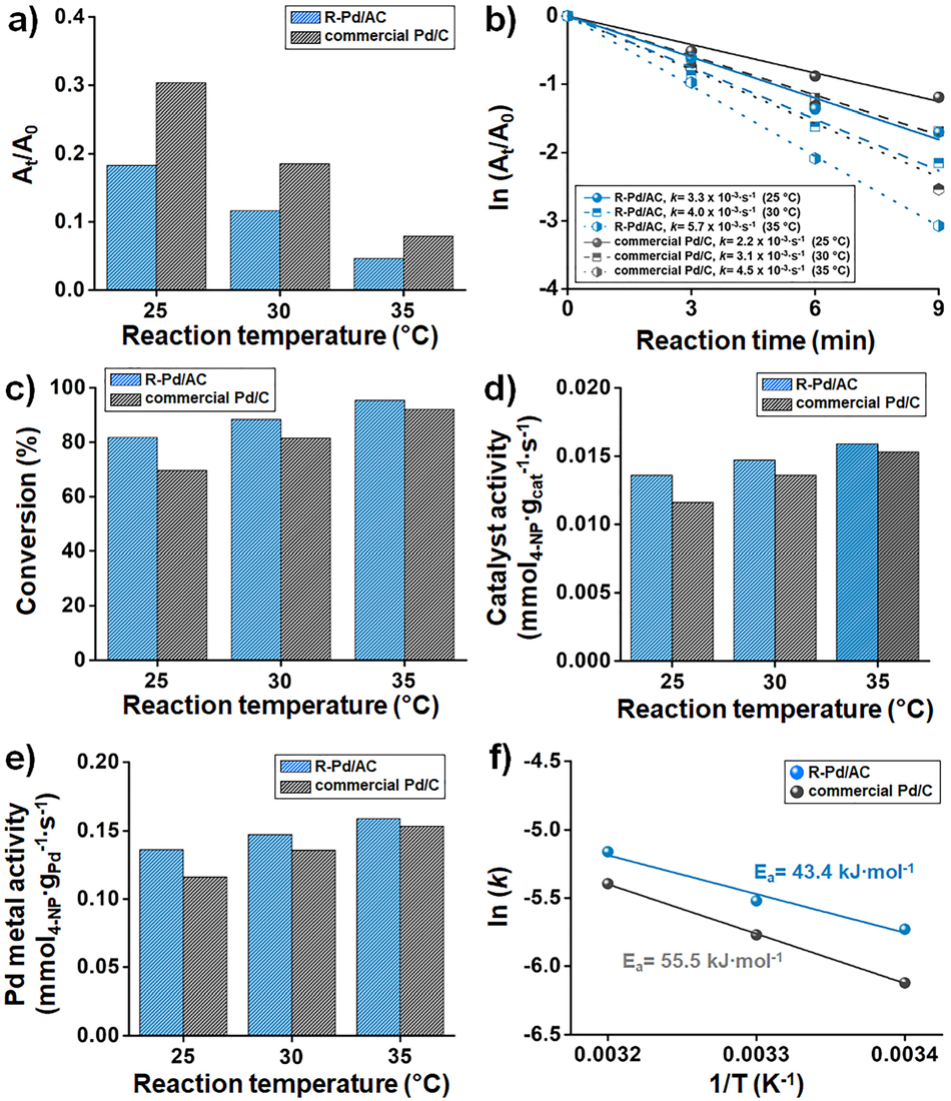
Temperature-dependent evaluation of catalytic performance for R-Pd/AC and commercial Pd/C in 4-NP reduction. (a) *A*_t_/*A*_0_ values at 9 min, indicating temperature-dependent reaction progress. (b) Pseudo-first-order kinetic plots from ln(*A*_t_/*A*_0_) *versus* time at three temperatures. (c) 4-NP conversion at 25 °C, 30 °C, and 35 °C, showing enhanced reactivity with increasing temperature. (d) Catalyst activity calculated at each temperature. (e) Pd metal activity for comparative assessment. (f) Arrhenius plots used to derive *E*_a_ values for R-Pd/AC relative to commercial Pd/C.

To investigate the effect of reaction temperature on catalytic performance, R-Pd/AC and commercial Pd/C catalysts were tested at 25 °C, 30 °C, and 35 °C using a custom-designed heating block integrated into the robotic platform. As the system periodically measured the temperature of the reaction solution and automatically adjusted the heater output in real time, the reaction temperature remained at the set value. As expected, the extent of conversion increased with temperature for both catalysts, while the corresponding *A*_t_/*A*_0_ values at 9 min decreased, indicating faster reaction completion at higher temperatures (Fig. 5a). Notably, R-Pd/AC consistently exhibited lower *A*_t_/*A*_0_ values than commercial Pd/C at all temperatures, demonstrating its superior reduction performance. Pseudo-first-order kinetics were confirmed by plotting ln(*A*_t_/*A*_0_) *versus* time, from which the apparent rate constants were derived (Fig. 5b). The R-Pd/AC catalyst exhibited *k* values of 3.25 × 10^−3^ s^−1^, 4.00 × 10^−3^ s^−1^, and 5.73 × 10^−3^ s^−1^ at 25 °C, 30 °C, and 35 °C, respectively, all of which were higher than those of commercial Pd/C under the same conditions. This trend was further reflected in the overall 4-NP conversion, where R-Pd/AC outperformed commercial Pd/C at every temperature point, achieving nearly complete conversion at 35 °C (Fig. 5c). Catalyst activity also increased with temperature and was consistently higher for R-Pd/AC compared to the commercial counterpart (Fig. 5d). When normalized by Pd content, the Pd metal activity showed a similar trend, suggesting more efficient utilization of accessible Pd active sites in R-Pd/AC (Fig. 5e). To determine the activation energy (*E*_a_) of each catalyst, the Arrhenius equation was applied by plotting ln(*k*) against the reciprocal of temperature (1/*T*, in Kelvin). The R-Pd/AC catalyst exhibited a lower *E*_a_ of 43.4 kJ mol^−1^, compared to 55.5 kJ mol^−1^ for commercial Pd/C (Fig. 5f), indicating a more kinetically favorable reaction pathway. This reduced energy barrier is likely attributable to the improved dispersion, smaller particle size, and higher accessibility of catalytically active sites in the R-Pd/AC catalyst.

### High-throughput screening and system scalability

To validate the throughput, sensitivity, and reproducibility of the automated platform, we screened 24 nanocatalysts at 25 °C: 22 metal-added Pd/AC (M-Pd/AC, where M = Na, Fe, Cu, Zn, Sn, *etc.*), a reference Pd/AC (R-Pd/AC), and commercial Pd/C. All M-Pd/AC samples were prepared *via* a one-pot co-impregnation method, maintaining a fixed metal additive ratio of 10 mol% relative to Pd for compositional consistency. The entire screening was conducted sequentially using a single robotic jig under fully automated conditions.

**Fig. 6:**
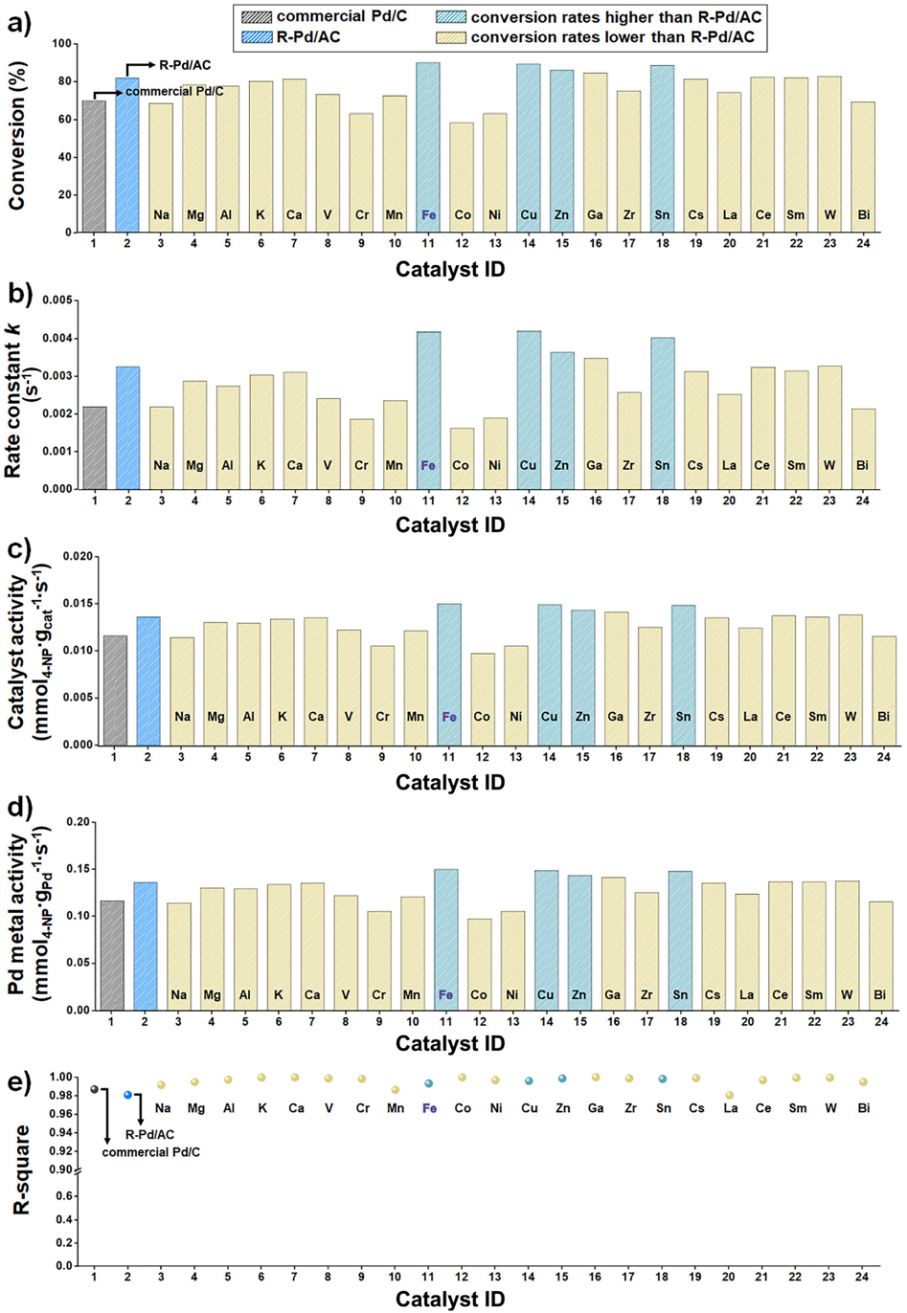
High-throughput screening results of the M-Pd/AC nanocatalyst library in the 4-NP reduction reaction, compared to R-Pd/AC and commercial Pd/C. (a) Conversion (%) measured after 9 min of reaction for each catalyst. (b) Pseudo-first-order rate constants (*k*) derived from ln(*A*_t_/*A*_0_) *versus* time plots. (c) Catalyst activity normalized by catalyst mass. (d) Pd metal activity normalized by Pd content. (e) Coefficient of determination (*R*^2^) values for kinetic fits.

Catalytic performance, assessed by 4-NP conversion, exhibited substantial variation depending on the identity of the additive (Fig. 6a). Fe-, Cu-, Zn-, and Sn-modified catalysts showed markedly enhanced activity, with Fe-Pd/AC achieving the highest conversion (89.8%). In contrast, additives such as Ni and Cr suppressed catalytic activity, suggesting adverse effects on the reaction process.

To ensure that these activity trends were not influenced by run-to-run variability, three independent replicate experiments (*n* = 3) were conducted for all 24 catalysts. The resulting conversion values reproduced the same performance ranking observed in Fig. 6a, confirming the high reproducibility and consistency of the automated workflow (Fig. S11). These performance trends reflect the dual impact of electronic and geometric modifications introduced by each additive. For instance, Fe is known to increase the electron density around Pd atoms, which facilitates hydride transfer during the reduction of 4-NP, thereby accelerating catalytic turnover.^42^ Cu and Zn, when incorporated into the Pd lattice, are reported to geometrically isolate neighboring Pd atoms and introduce local lattice strain. This perturbation enhances metal dispersion and modulates the spatial distribution of active sites, effectively increasing the number of accessible and selectively reactive centers.^43^ On the other hand, certain additives may impair catalytic performance by altering the electronic environment of Pd or introducing structural features that hinder access to active sites.^44^ These effects can reduce the availability or reactivity of Pd centers, ultimately leading to lower catalytic activity. The corresponding *k* values closely reflected the observed conversion trends (Fig. 6b).

To complement the automated kinetic measurements and clarify the structural origins of catalytic performance, selective characterization was performed on the commercial Pd/C catalyst, the synthesized R-Pd/AC reference catalyst, and two representative M-Pd/AC samples: a high-performing Fe-Pd/AC and a low-performing Co-Pd/AC. Transmission Electron Microscopy (TEM) revealed that the commercial Pd/C sample contained a broad and heterogeneous particle-size distribution, with numerous particles exceeding 30 nm (Fig. S12a). In contrast, the synthesized R-Pd/AC catalyst exhibited narrower distribution, with Pd nanoparticles uniformly dispersed at an average size of approximately 20 nm (Fig. S12b and c). X-ray Diffraction (XRD) analysis confirmed well-defined Pd diffraction peaks, and Scherrer analysis of the (111) reflection yielded a crystallite size of 20 nm, consistent with the TEM observations (Fig. S12d).

For the high-performing Fe-Pd/AC catalyst, High-Angle Annular Dark-Field Scanning Transmission Electron Microscopy (HAADF-STEM) and elemental mapping showed uniformly dispersed Pd nanoparticles and Fe species that were closely associated with the Pd domains rather than forming separate clusters (Fig. S13a–e). XRD patterns showed no distinct Fe-containing crystalline phases, and the Pd crystallite size was comparable to that of R-Pd/AC (Fig. S13f). In contrast, the low-performing Co-Pd/AC catalyst displayed larger Pd nanoparticles (greater than 30 nm) with a broad size distribution, and the Co component formed segregated Co-rich domains that were spatially separated from the Pd particles, as confirmed by elemental mapping (Fig. S14). This morphology suggests that Co addition disrupts uniform Pd nucleation, leading to heterogeneous metal distribution and partial aggregation.

These structural distinctions correlate closely with the catalytic trends observed across the M-Pd/AC library. Homogeneous additive distribution enhances Pd dispersion and modulates the local electronic environment, which collectively facilitate efficient electron transfer during the 4-NP reduction reaction.

Catalysts with Fe, Cu, Zn, and Sn again stood out with significantly higher *k* values, confirming accelerated reaction kinetics. These kinetic enhancements were further validated by catalyst activity measurements normalized to catalyst mass (Fig. 6c), and Pd metal activity normalized to Pd content (Fig. 6d), both of which followed similar trends, highlighting not only improved overall reactivity, but also more effective utilization of Pd active sites in these systems.

To assess the reliability of the kinetic analysis, the goodness-of-fit (*R*^2^) values for the pseudo-first-order plots were examined across all catalysts (Fig. 6e). *R*^2^ values exceeding 0.95 indicate that the data conform well to the pseudo-first-order kinetic model and that the UV-Vis measurements were internally consistent. However, *R*^2^ reflects only the goodness of fit and does not guarantee absolute accuracy. In practice, *R*^2^ can vary with spectral quality or baseline stability, and *k* value is highly sensitive to the catalyst-to-reactant ratio, meaning that even small differences in catalyst loading can lead to noticeable shifts in the extracted rate constant. To contextualize the platform's accuracy, literature-reported rate constants for commercial Pd/C in the NaBH_4_-mediated reduction of 4-nitrophenol were surveyed, and the *k* values obtained in this study fell within the expected ranges under comparable reaction conditions.^45,46^

All kinetic metrics including absorbance spectra, conversion, rate constant, and both catalyst- and Pd-specific activity were analyzed in real time using a custom-developed data interface (Fig. S15–S17). This automated platform allowed high-throughput extraction and visualization of key reaction parameters across all 24 catalyst variants. By integrating multiple performance indicators into a single dashboard, the system enabled rapid and quantitative comparison among catalysts, facilitating precise ranking and identification of promising candidates. Such streamlined analysis greatly accelerated data-driven decision-making for additive selection and formulation optimization, ultimately enhancing the efficiency of the catalyst discovery pipeline.

**Fig. 7:**
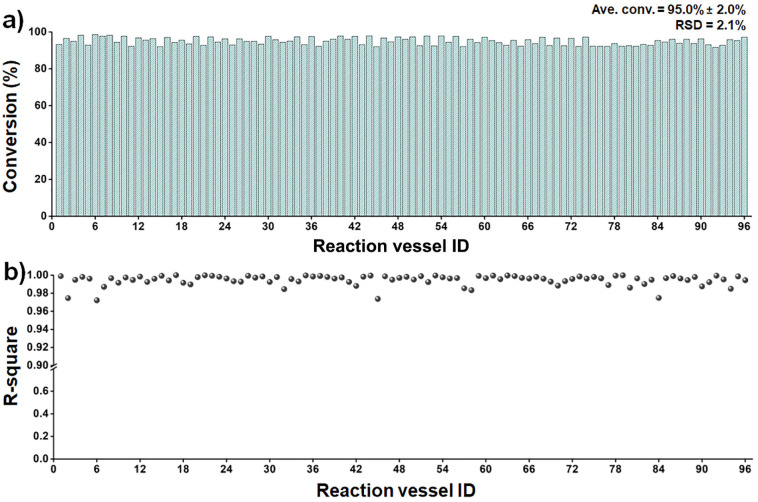
Reproducibility evaluation of 96 Fe-Pd/AC nanocatalyst samples using fully automated and unattended robotic operation. (a) Conversion profiles across 96 independent measurements, demonstrating consistent catalytic performance. (b) *R*^2^ values derived from pseudo-first-order rate constant (*k*) fittings.

We evaluated the scalability and robustness of the system through 96-sample high-throughput screenings at 30 °C. These tests employed Fe-Pd/AC synthesized at a larger batch scale, which had been identified in preliminary evaluations as the top-performing catalyst (Fig. 7a). The scaled-up synthesis provided sufficient and consistent material for repeated assessments. Across all runs, conversion values remained highly consistent (95.0% ± 2.0%), and kinetic fittings yielded *R*^2^ values close to unity (Fig. 7b), demonstrating excellent reproducibility in robotic operation, temperature control, and UV-Vis measurements. The complete evaluation of 96 samples was accomplished within 16 h 40 min under fully automated conditions, averaging approximately 10 min per sample (Movie S3). This capability enabled unattended overnight operation and facilitated the rapid identification of optimal candidates.

**Fig. 8:**
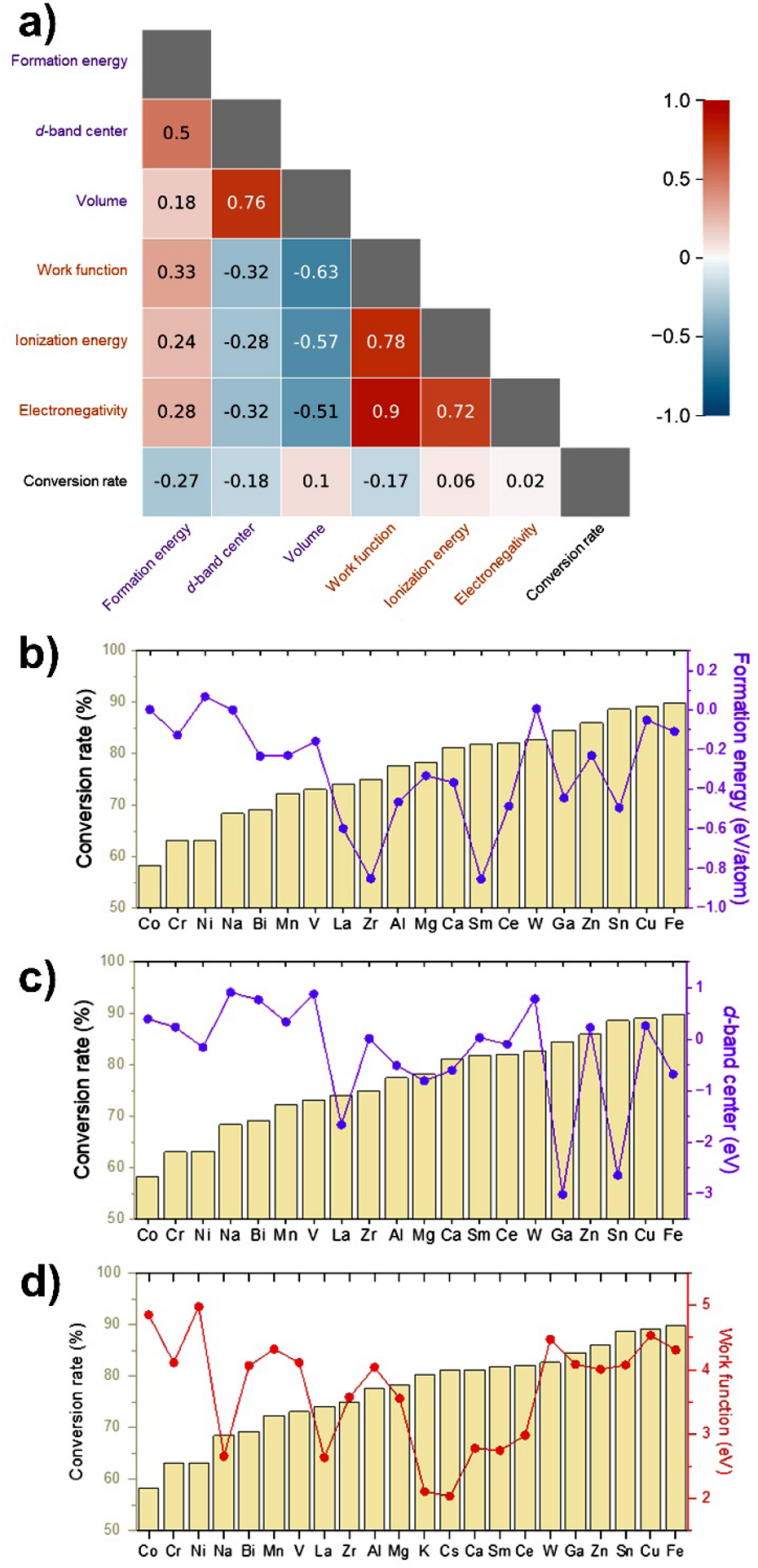
(a) Correlation plot of the conversion rates of M-Pd/AC catalysts with material properties extracted from the Materials Project database and the elemental properties of the M metals. Violet labels denote M-Pd alloy systems, and orange labels denote unary M metals. Correlation coefficients were calculated excluding M = Cs and M = K, due to the absence of data for the Cs-Pd system and the presence of only a K-rich phase for the K-Pd system in the database. (b) Formation energies, (c) d-band center values, and (d) work functions of the M metals, plotted in order of increasing M-Pd/AC conversion rate, which is shown as background bars.

To investigate the correlation between the catalytic conversion rate of M-Pd/AC and relevant material properties, correlation coefficients were calculated using DFT data for M-Pd alloy and unary M metal systems obtained from the Materials Project database.^47^ For M-Pd alloys, the most stable phases with compositions closest to the experimental additive ratio (10 mol%) were selected. For unary M metals, the most stable phases were chosen to obtain weighted work function values. Additionally, the elemental ionization energy and Pauling electronegativity of the additive metals were considered.^48,49^ Although the evaluated material properties generally exhibited weak correlations with the experimental conversion rates, the formation energy of M-Pd alloys showed the strongest negative correlation (−0.27), followed by the d-band center (−0.18) and the work function of the additive metals (−0.17) (Fig. 8a). These results suggest that more stable M-Pd alloy formation suppresses phase separation of the additive metals, thereby enhancing catalytic activity (Fig. 8b). Lower d-band centers and lower work functions also showed a weaker but consistent trend toward improved performance (Fig. 8c and d).

Notably, certain metals such as Fe, Cu, and Zn demonstrated higher experimental performance than suggested by the DFT-derived descriptors. This underestimation likely arises from nanoscale effects not captured by bulk-level calculations, including enhanced Pd dispersion, favorable surface alloying, and synergistic electronic-geometric modifications that facilitate hydride transfer during 4-NP reduction. Conversely, metals such as La and Sm appeared favorable according to the descriptor values but showed relatively low experimental performance. This overestimation may result from surface oxide formation, incomplete alloying at the nanoparticle scale, or geometric blocking of active sites, all of which can reduce catalytic efficiency despite favorable bulk electronic properties.

These findings highlight that while bulk-level DFT descriptors can capture general performance trends, experimental validation remains essential to account for surface chemistry, nanostructure, and synthesis-dependent effects. The complete dataset supporting this correlation analysis, including all numerical values and graphical comparisons, is provided in the SI (Table S2 and Fig. S18).

## Conclusions

We developed a fully automated robotic platform for high-throughput, reproducible, and data-rich screening of Pd-based nanocatalysts with minimal human intervention. Integrating two synchronized robotic arms, automated liquid handling, and real-time UV-Vis spectroscopic monitoring, the system autonomously completes 96-sample screening cycles within 16 h 40 min, enabling unattended overnight operation. Using the 4-NP to 4-AP reduction as a model reaction, we evaluated 24 catalysts, including 22 metal-added Pd/AC variants, a reference Pd/AC (R-Pd/AC), and a commercial Pd/C catalyst. Additives such as Fe, Cu, Zn, and Sn markedly enhanced catalytic performance, whereas Ni, Cr, Bi, and Co showed minimal or adverse effects. Correlation analysis with Materials Project DFT data revealed that the formation energy of M-Pd alloys exhibited the strongest negative correlation with conversion rate, followed by the d-band center and the work function of additive metals. Stable alloy formation likely suppresses phase separation, while a lower d-band center and reduced work function promote more favorable electron transfer at active sites. These descriptors accounted for the high activity of Fe, Cu, and Zn, but overpredicted the performance of La and Sm, underscoring the importance of considering surface chemistry, nanostructure, and synthesis-dependent effects alongside bulk electronic properties. This modular and scalable platform demonstrates excellent reproducibility, adaptability to large-batch catalyst synthesis, and compatibility with diverse catalytic reactions and analytical techniques. By coupling automated experimentation with real-time data analysis, intersample comparison, and automated performance ranking, the system provides a robust infrastructure for catalyst discovery. Its potential integration into closed-loop, AI-guided workflows opens new opportunities to accelerate rational catalyst design across a wide range of chemical transformations.

## Author contributions

Shin Wook Kang: writing – original draft; software – robotic control system implementation; investigation – automated screening experiments. Kyung Hee Oh: investigation – nanocatalyst synthesis; formal analysis – spectroscopic measurements. Kanghoon Yim: data curation – materials project data extraction; formal analysis. Sanha Jang: software – system integration support; data curation; formal analysis. Jin Gyu Lee: investigation – experimental execution; data curation – data collection. Jung-Il Yang: visualization; investigation. Ji Chan Park: conceptualization; methodology – automated workflow design; supervision; writing – review & editing; manuscript finalization with input from all authors.

## Conflicts of interest

There are no conflicts to declare.

## Supplementary Material

SC-OLF-D5SC06192J-s001

SC-OLF-D5SC06192J-s002

SC-OLF-D5SC06192J-s003

SC-OLF-D5SC06192J-s004

SC-OLF-D5SC06192J-s005

SC-OLF-D5SC06192J-s006

## Data Availability

Supplementary information (SI): all supporting data, including images, videos, tables, and detailed descriptions of experimental procedures, are available in the SI. See DOI: https://doi.org/10.1039/d5sc06192j.
