# Supplementary material for: Fully automated and high-fidelity robotic platform enabling accelerated discovery of nanocatalysts
Source: Chem Sci. 2025 Dec 30;17(9):4659–68. doi: 10.1039/d5sc06192j (PMC12794936; doi:10.1039/d5sc06192j)
Supplement: SC-017-D5SC06192J-s006 [file SC-017-D5SC06192J-s006.pdf]

Please do not adjust margins

Electronic Supplementary Information

Fully Automated and High-Fidelity Robotic Platform Enabling Accelerated Discovery of Nanocatalysts

Shin Wook Kang,[a§] *Kyung Hee Oh,*[a§] *Kanghoon Yim,*[b] *Sanha Jang,*[a] *Jin Gyu Lee,*[a] *Jung-Il Yang,*[a] *and Ji Chan Park*[a,c]*

[a] Clean Fuel Research Laboratory, Korea Institute of Energy Research, Daejeon 34129, Republic of Korea.

[b] Energy AI & Computational Science Laboratory, Korea Institute of Energy Research, Daejeon 34129, Republic of Korea.

[c]Energy Engineering, University of Science and Technology, Daejeon 34113, Republic of Korea

§ S.W.K. and K.H.O. contributed equally to this work.

*Corresponding Author (email): jcpark@kier.re.kr

Please do not adjust margins

Please do not adjust margins

Experimental Section

Chemicals. Palladium(II) acetylacetonate ($Pd(acac)_2$, 99 %), iron(II) acetate ($Fe(CH_3CO_2)_2$, 99 %), activated charcoal (100 mesh), 4-nitrophenol (4-NP, ≥97 %), sodium borohydride ($NaBH_4$, ≥96 %), and various metal precursor salts in Table S1 were purchased from Sigma-Aldrich and used as received. Ethanol (99.9 %) was supplied by Samchun Chemicals. A commercial Pd/C catalyst (Evonik NOBLYST® P1070, 10 wt% Pd) was used as a reference.

Synthesis of R-Pd/AC and M-Pd/AC nanocatalysts. R-Pd/AC was prepared with a nominal Pd loading of 10 wt%. In a 1 mL glass vial, 2.3 mg of $Pd(acac)_2$ (7.55 μmol) and 7.2 mg of activated charcoal (0.6 mmol) were dispersed in 100 μL of ethanol. For M-Pd/AC variants, the same amounts of $Pd(acac)_2$ and activated charcoal were first suspended in 50 μL of ethanol. A concentrated stock solution of the additive metal precursor (10 mol% relative to Pd) was prepared by dissolving it in 2.5 mL of ethanol (corresponding to a 50× scale). Then, 50 μL of this stock solution was added to the Pd/AC slurry. The resulting mixture was vortexed and sonicated for 30 min to ensure uniform dispersion. All slurries were then transferred to alumina crucibles and reduced under hydrogen flow (200 mL·min^{-1}) in a tubular furnace. The temperature was ramped to 400 °C at a rate of 3.75 °C·min^{-1}, and maintained for 30 min. After cooling under nitrogen, the reduced catalysts were collected for testing.

Scale-up synthesis of Fe-Pd/AC catalyst. Fe-Pd/AC was also synthesized on a larger scale (×139 batch scale) for extended testing. A total of 1.0 g of activated charcoal (83.3 mmol) and 0.318 g of $Pd(acac)_2$ (1.04 mmol) were premixed in a high-energy ball mill at 1750 rpm for 5 min using a methacrylate grinding ball. Separately, 18.2 mg of $Fe(CH_3COO)_2$ (104.6 μmol, 10 mol% relative to Pd) was dissolved in 13.9 mL of ethanol and added to the solid mixture. After stirring, the slurry was dried at 50 °C under vacuum for 1 h, followed by reduction at 400 °C for 30 min under a hydrogen flow. The resulting product was cooled under nitrogen and collected for testing.

Catalyst reaction preparation for robotic evaluation. Twenty-four catalyst samples (R-Pd/AC, commercial Pd/C, and 22 M-Pd/ACs) were each manually weighed to a nominal mass of 8.00 mg into single-use polyethylene bottles using a high-precision microbalance (±0.005 mg tolerance). For all subsequent kinetic analyses, the nominal target mass was used, as such small deviations were negligible with respect to the calculated catalytic activity. Using an Opentrons OT-2 liquid-handling robot, 1.0 mL of 4-NP stock solution (0.072 M) was dispensed into each bottle (**Movie S4**†). The bottles were then placed in a custom-designed shaker jig. An auxiliary robot (Wlkata Mirobot), paired with a peristaltic pump, delivered 38 mL of deionized water into each bottle along automated, preprogrammed motion paths (**Movie S5**†). The reducing agent solution was prepared by dissolving 1.0 g of $NaBH_4$ and 2.0 g of NaOH in 125 mL of deionized water, yielding a pH of approximately 12 to ensure reductive stability. The solution was subsequently loaded into a reservoir located on the robotic platform.

Automated UV-Vis measurement protocol. After the experimental conditions are set, robot 1 initiates operation via pre-installed robot control program 1. Following the initial manual setup described above, all subsequent experimental steps were executed automatically without human intervention. Once activated, the robots are automatically synchronized with the controller, initiating all movement sequences, data logging, and process execution without manual intervention. The status of the catalytic reactions and data acquisition is displayed in real time and can also be remotely monitored via a CCTV system. To ensure consistent reaction kinetics and homogeneous dispersion, the system employs controlled orbital shaking at 200 rpm, which is sufficient to achieve uniform mixing without causing liquid overflow from the reaction bottles. In addition, temperature regulation and reaction homogeneity are achieved through the use of custom-designed aluminum heating blocks and integrated thermocouples. These thermally conductive blocks maintain the desired reaction temperature uniformly across all bottles, while orbital shaking further ensures dispersion of reactants and catalysts. Prior to initiating the reaction, the solution inside each bottle was fully equilibrated to the set temperature, and thermocouple-based measurements

 This journal is © The Royal Society of Chemistry 20xx

Please do not adjust margins

Please do not adjust margins

confirmed that the reaction temperature remained stable throughout the reaction period due to the feedback-controlled heating system.

To investigate the effect of temperature on catalytic performance, catalysts were tested at 25 °C, 30 °C, and 35 °C using the integrated heating system within the robotic platform. Prior to initiating the catalytic reaction, blank measurements are performed to establish a baseline for UV-Vis absorbance. Robot 1 retrieves a clean UV-Vis cell from the storage rack and sequentially dispenses 1.0 mL of deionized water twice into the cell (total volume: 2.0 mL). The cell is then placed into the spectrometer jig, and a blank scan is conducted using the UV-Vis spectrometer, controlled by robot control program 1. After measurement, the UV-Vis cell used for the blank is automatically discarded and replaced by robot 1. To initiate the reaction, robot 2 (operated by robot control program 2) collects 1.0 mL of $NaBH_4$ solution using a fresh pipette tip and dispenses it into the designated reaction bottles. The used tip is discarded immediately after injection to prevent contamination. The bottle is then gently shaken to ensure thorough mixing of the reactants. Robot 1 subsequently aspirates 60 μL of the reaction mixture at each predetermined time point (0, 3, 6, and 9 min), transfers the aliquot into a fresh UV-Vis cell prefilled with 2.0 mL of deionized water, thoroughly mixes the contents, and places the cell into the UV-Vis spectrometer. Each time-point measurement is performed on an independently sampled aliquot drawn directly from the ongoing reaction, ensuring accurate tracking of reaction kinetics over time. This fully automated sequence is executed for 24 samples per cycle. Upon completion of each cycle, robot 2 replaces the UV-Vis cell jig and pipette tips to ensure cleanliness and prevent cross-contamination before processing the next batch. All acquired absorbance data were automatically transmitted to the control PC for real-time processing and storage, completing the fully automated catalytic activity screening workflow under the coordination of robot control programs 1 and 2. All robotic components in the automated platform including both commercially available instruments and custom-fabricated parts are summarized in Table S3†.

Characterization. Transmission electron microscopy (TEM) was performed using a JEOL F200 microscope operated at 200 kV and equipped with an energy-dispersive X-ray spectroscopy (EDS) detector (JED-2300T, JEOL Ltd.). TEM samples were prepared via a conventional drop-casting method, where several drops of the colloidal suspension were deposited onto lacey carbon-coated copper grids (Ted Pella, Inc.) and dried under ambient conditions. X-ray diffraction (XRD) measurements were conducted using a high-power powder diffractometer (Rigaku D/MAX-2500, 18 kW) with Cu Kα radiation. Data were collected over a 2θ range of 20-80° with a scanning speed of 3° min^{-1} and a step size of 0.01°.

This journal is © The Royal Society of Chemistry 20xx

Please do not adjust margins

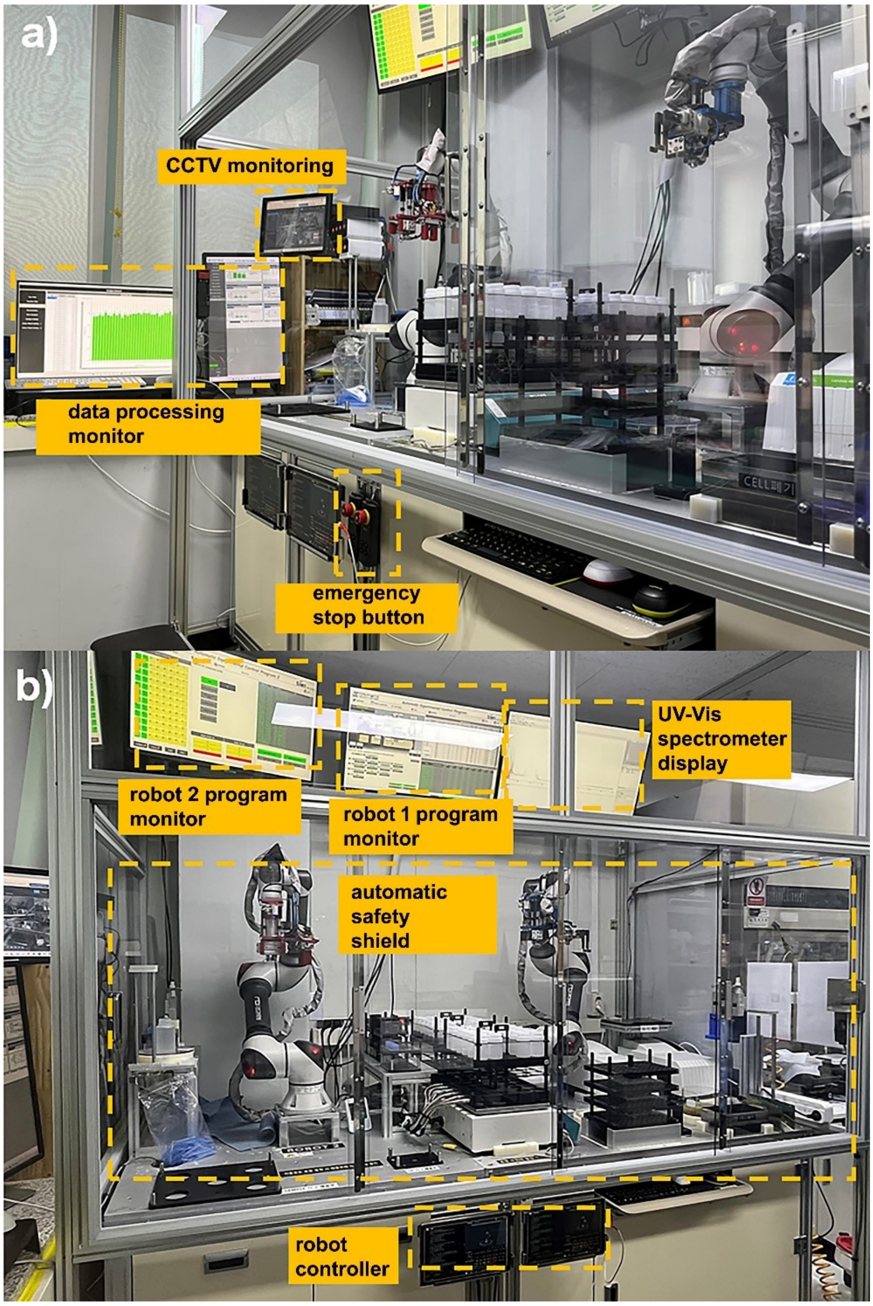

Figure S1. Overview photographs of the automated catalyst evaluation system. (a) Side view of the robot-based high-throughput catalyst screening platform, featuring a CCTV monitoring system, data-processing monitor, emergency stop button, and two coordinated robotic arms operating within a sealed enclosure for operational safety and precision. (b) Front view of the full system layout, showing the two robotic arms controlled via independent program monitors, an integrated UV-Vis spectrometer for in-line kinetic measurements, and a centralized controller module housed beneath the automatic safety shield for coordinated execution of multistep workflows.

This journal is © The Royal Society of Chemistry 20xx

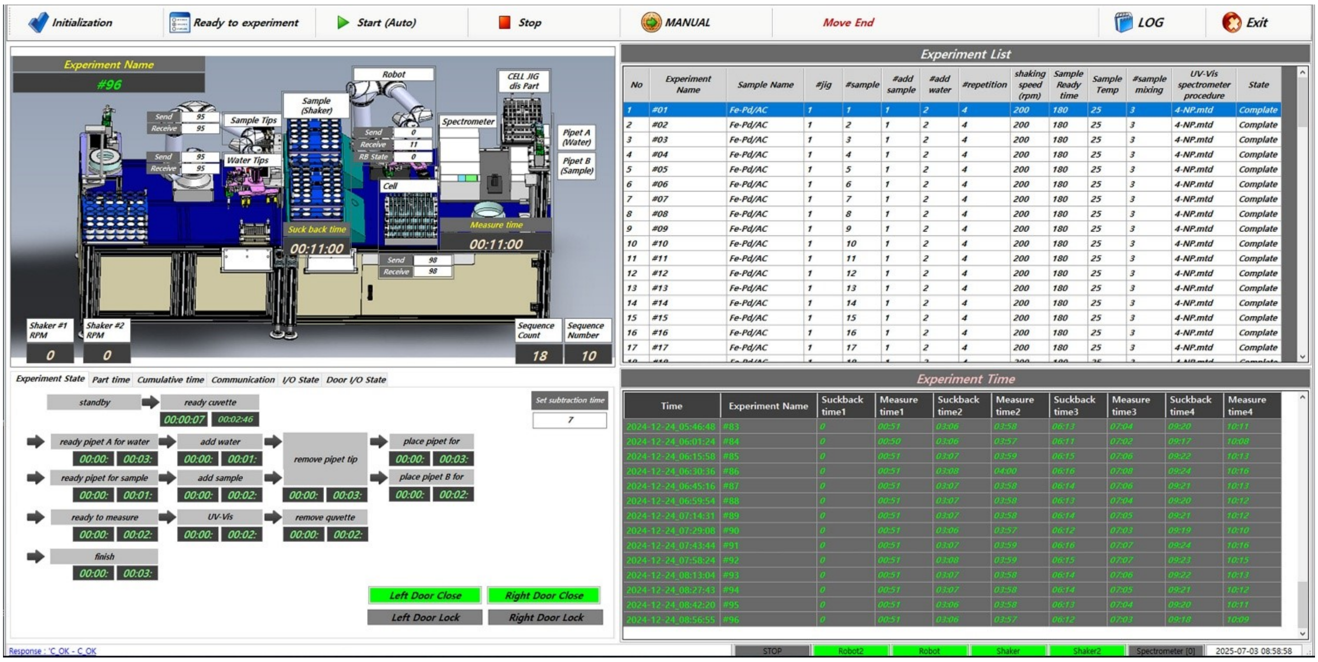

Figure S2. Graphical user interface (GUI) of the automated catalyst screening platform used in robot control program 1. The central interface displays the real-time operation of the system, including robotic handling, pipetting, UV-Vis measurements, and sample shaking. A schematic diagram shows the status of each module, including robot arm positioning, spectrometer timing, shaker speed (RPM), and jig state. The right-side panel lists experimental details for each sample, such as composition, repetition, shaking speed, and UV-Vis procedure. The bottom panel tracks detailed time logs for every measurement and pipetting cycle, enabling transparent, time-resolved diagnostics and process control across all 96 samples. This GUI provides intuitive monitoring and precise control of automated workflows assigned to robot program 1.

Please do not adjust margins

Please do not adjust margins

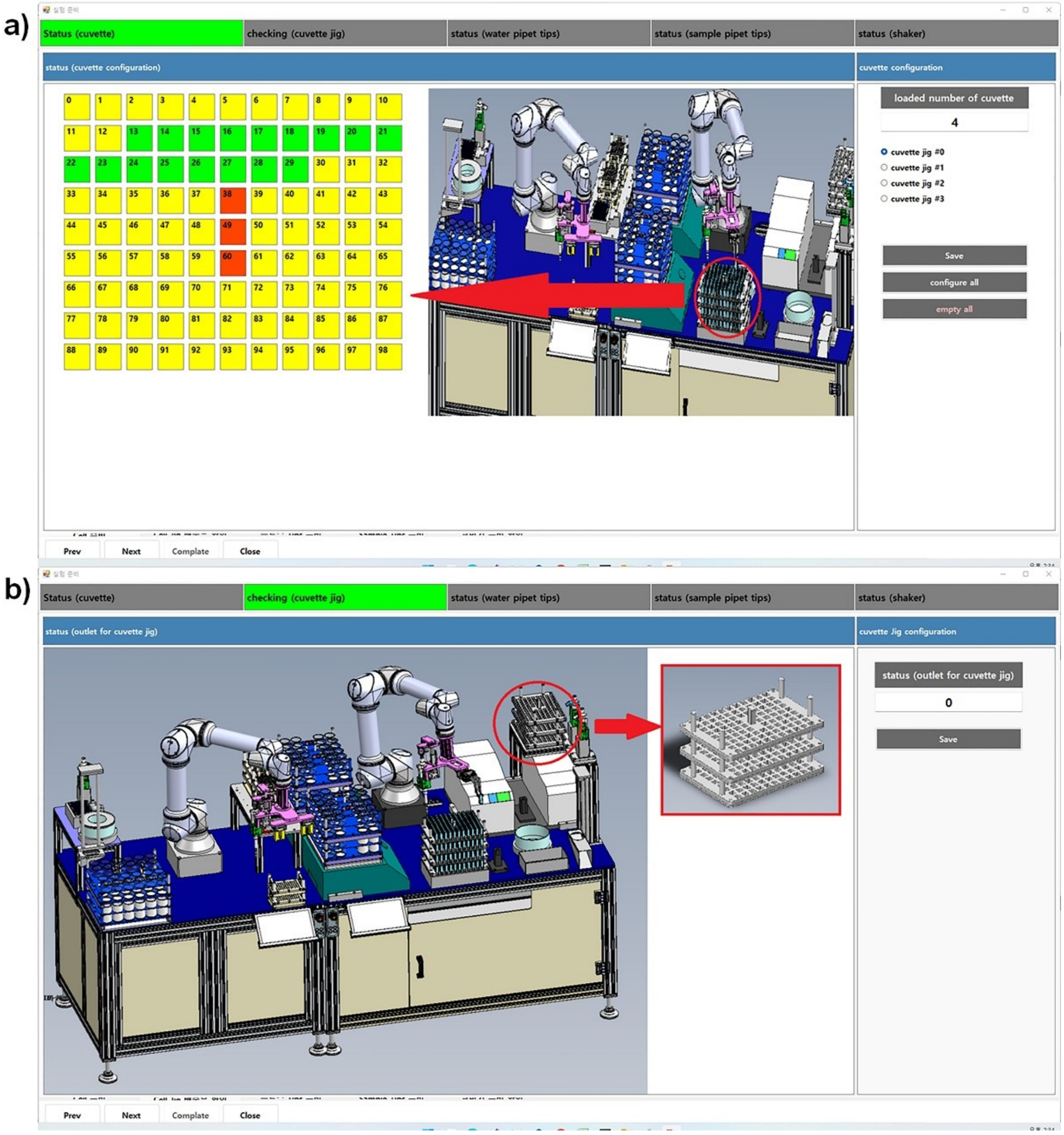

Figure S3. Graphical interface for UV-Vis cell management in the automated system used in robot program 1. (a) The interface displays the real-time status of disposable UV-Vis cells within a 96-well configuration, color-coded by usage: green for occupied cells, yellow for empty, and red for unused slots. The schematic inset shows the physical layout of the cuvette loading module as operated in robot program 1. (b) A 3D visualization highlights the cuvette outlet jig, where used cells are discarded after measurement. The right panel shows a magnified view of the outlet tray, allowing users to confirm the final disposal state. This interface is central for visualizing and validating cuvette handling during automated workflows executed by robot program 1.

This journal is © The Royal Society of Chemistry 20xx

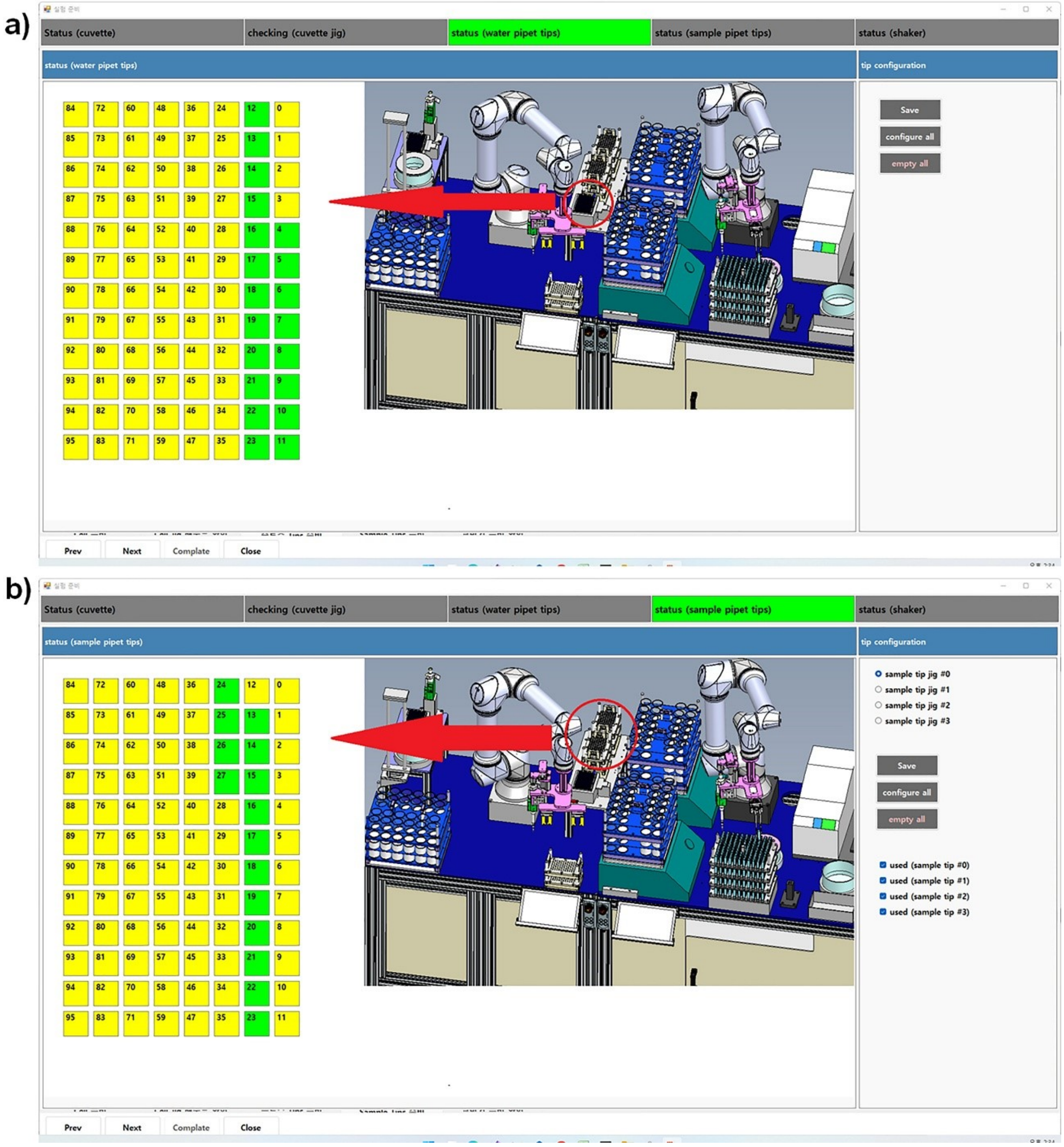

Figure S4. Pipette tip management interface for automated reagent handling in robot program 1. (a) The system monitors the status of water pipette tips using a 96-slot grid, where green indicates available tips and yellow indicates used slots. The inset highlights the physical location of the water tip tray on the robotic platform operated under robot program 1. (b) Similarly, the interface tracks sample pipette tips with real-time updates, distinguishing between filled (green) and empty (yellow) tips. The inset shows the location of the sample tip tray used for efficient sample handling during automated runs executed by robot program 1.

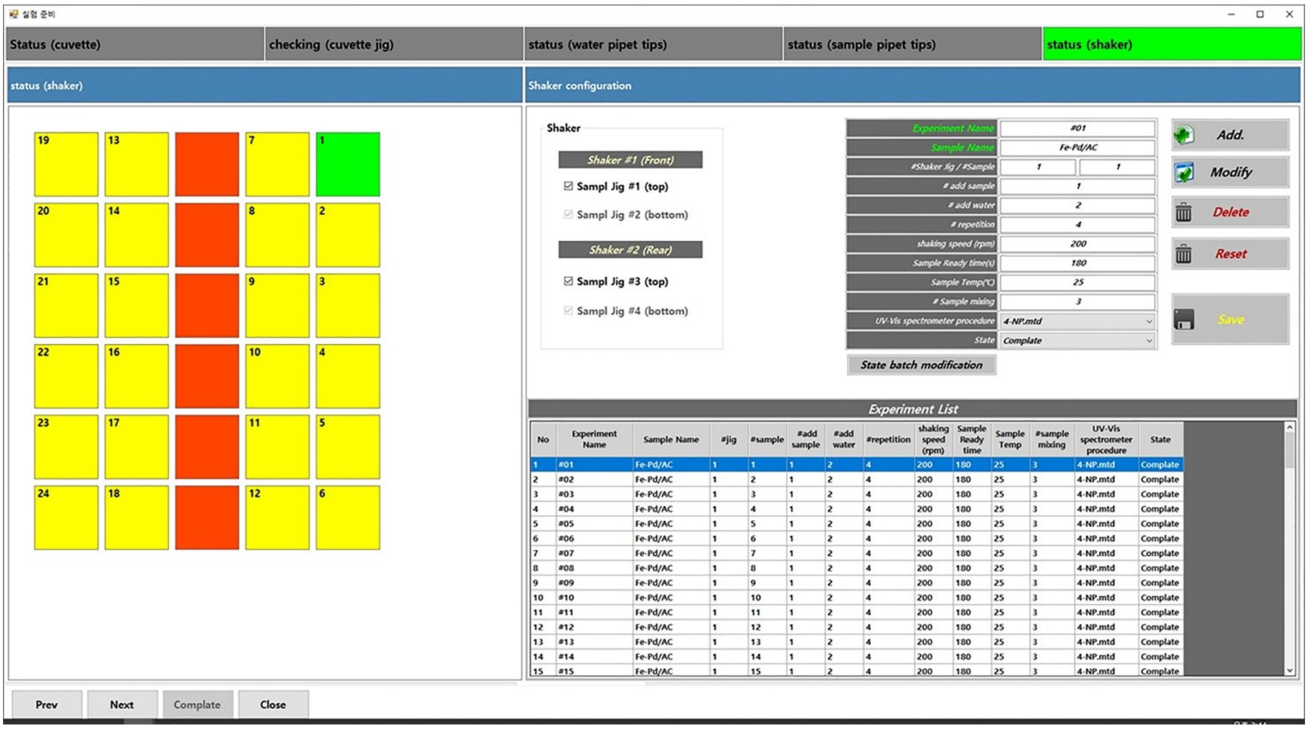

Figure S5. Shaker configuration interface for automated catalyst preparation in robot program 1. The left panel visualizes the current status of the shaker tray, with green squares indicating positions filled with disposable UV-Vis cells, yellow indicating empty cells, and red representing unused positions. The right panel displays programmable parameters for each experiment, including sample ID, jig assignment, reagent volumes, shaking speed, reaction temperature, and mixing cycles-all executed under robot program 1. The bottom panel lists completed experiments for batch tracking and state management during automated workflows controlled by robot program 1.

This journal is © The Royal Society of Chemistry 20xx

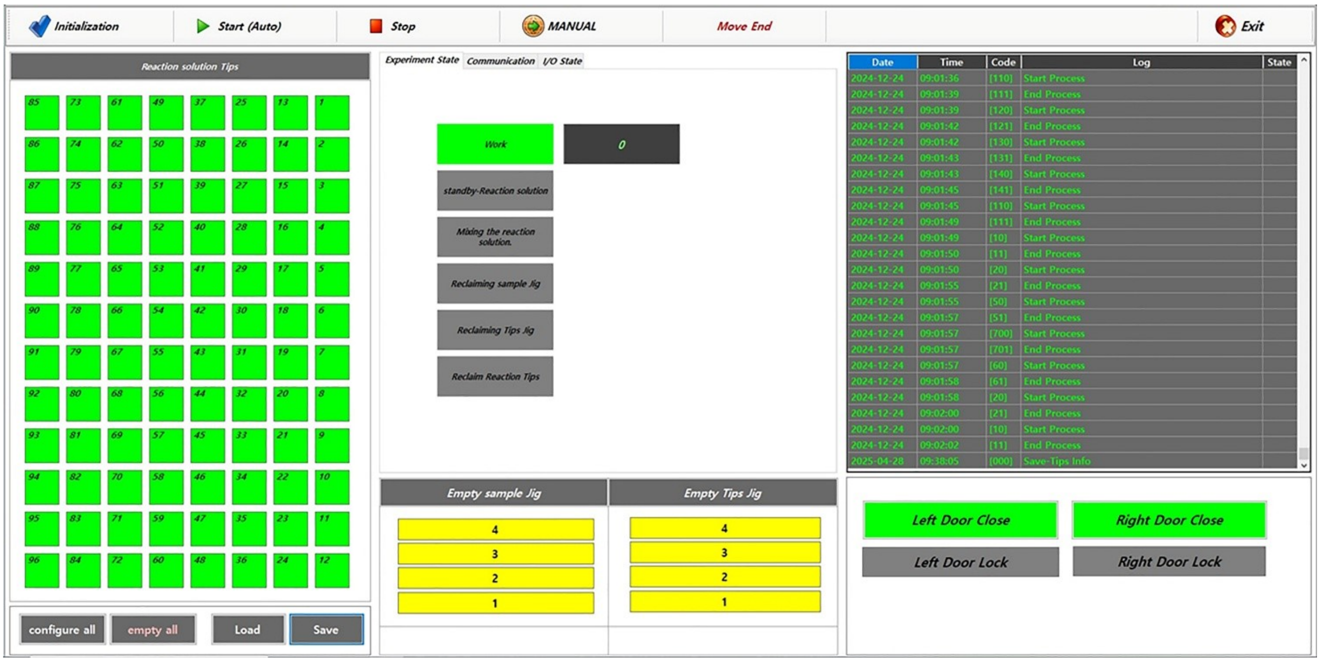

Figure S6. Status interface for reaction solution pipetting and system operations in robot program 2. The left panel shows the availability of 96 disposable pipette tips for reaction solution dispensing, with green indicating that all tips are filled and ready. The center panel tracks the current operation state such as pipetting, jig reclaim, or mixing within the workflow of robot program 2. The right panel logs each time-stamped robotic event with associated operation codes, enabling detailed diagnostics and traceability. The bottom section displays the number of available empty jigs for samples and tips, supporting efficient resource management and uninterrupted experimental execution under robot program 2.

Please do not adjust margins

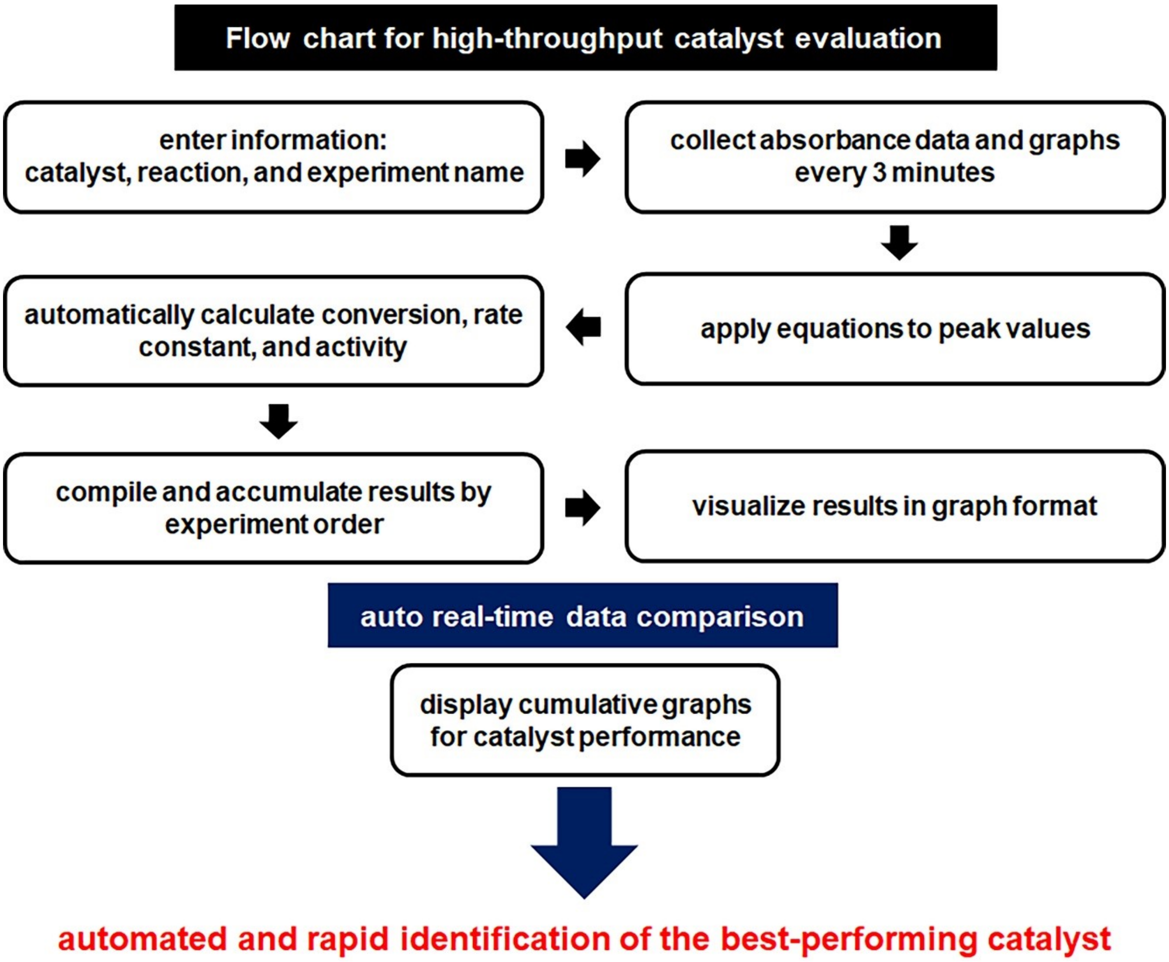

Figure S7. Flowchart of the automated high-throughput data analysis process for catalyst screening. The software workflow begins with entering basic experiment details, such as catalyst identity, reaction type, and experiment name. Absorbance data are collected every 3 min and processed using predefined equations to calculate peak values. The system then automatically determines the conversion, rate constant, and activity, compiling the results in sequential order. All outputs are visualized as graphs and compared in real time, enabling rapid and automated identification of the best-performing catalyst based on cumulative performance metrics.

This journal is © The Royal Society of Chemistry 20xx

Please do not adjust margins

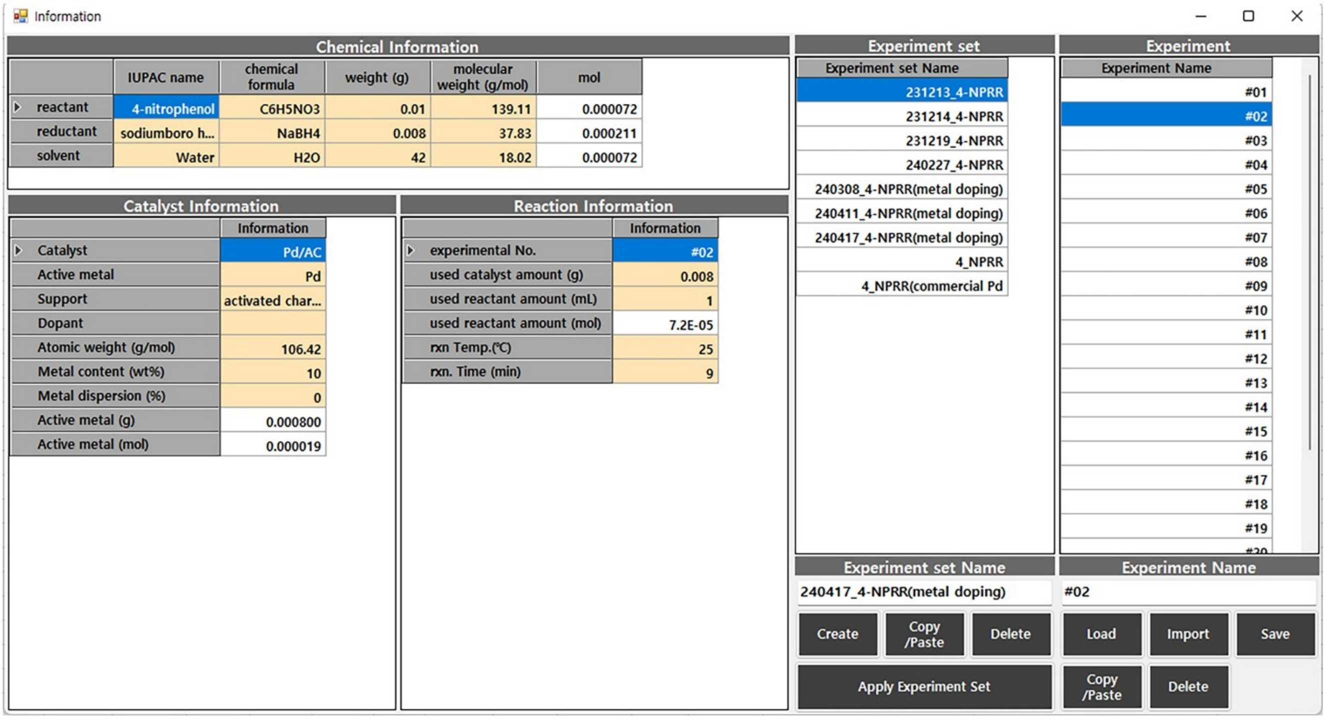

Figure S8. User interface for pre-input of experimental and catalyst information. The software interface enables users to enter essential chemical, catalyst, and reaction parameters before initiating automated experiments. Chemical information includes the identity, formula, mass, and molar amounts of the reactant (4-nitrophenol), reductant ($NaBH_4$), and solvent (DI water). Catalyst information includes metal type, support, atomic weight, metal loading, and amount of active metal. Reaction information includes catalyst and reactant quantities, reaction temperature, and time. These inputs ensure accurate kinetic calculations and reproducible catalyst evaluation results. Experiment set and individual experiment management functions are provided for streamlined batch operations.

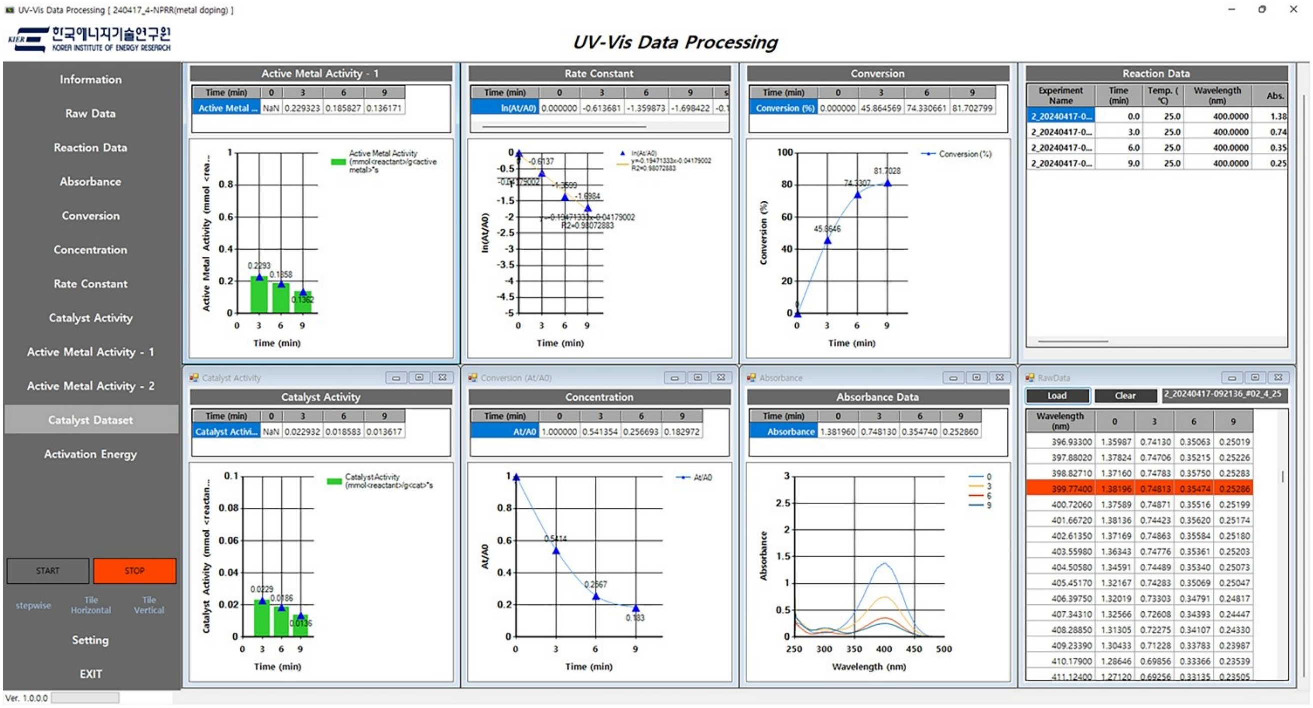

Figure S9. Screenshot of the UV-Vis data processing software used for kinetic analysis. The interface displays a comprehensive set of quantitative analyses for catalyst evaluation. Key metrics (absorbance, A_t/A_0, conversion (%), rate constant, catalyst activity, and active metal activity) are automatically calculated and plotted based on UV-Vis spectral data collected over time.

This journal is © The Royal Society of Chemistry 20xx

Please do not adjust margins

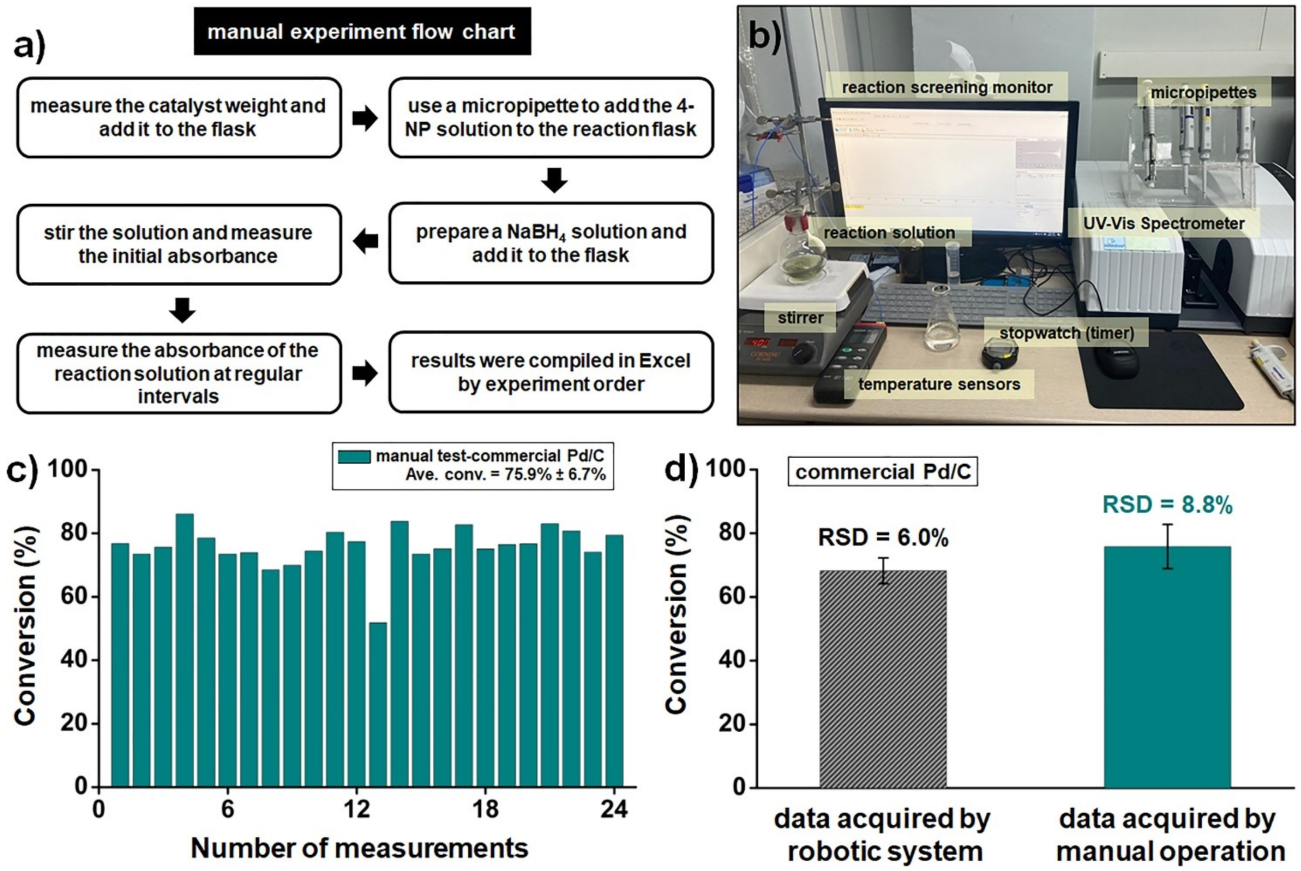

Figure S10. Comparative analysis of manual versus robotic catalyst evaluation workflows. (a) Flowchart of the manual test procedure for the reduction of 4-NP using commercial Pd/C, including catalyst weighing, reagent addition, and UV-Vis measurements at regular intervals. (b) Photograph of the manual experimental setup showing the reaction monitoring system, UV-Vis spectrometer, micropipettes, temperature sensor, and stopwatch for manual data acquisition. (c) Conversion results from 24 manually repeated experiments using commercial Pd/C. (d) Side-by-side comparison of conversion values obtained from robotic and manual operations for the same catalyst. Each data point corresponds to an independent replicate reaction (n = 24), carried out in separate reaction vessels prepared under identical experimental conditions.

Please do not adjust margins

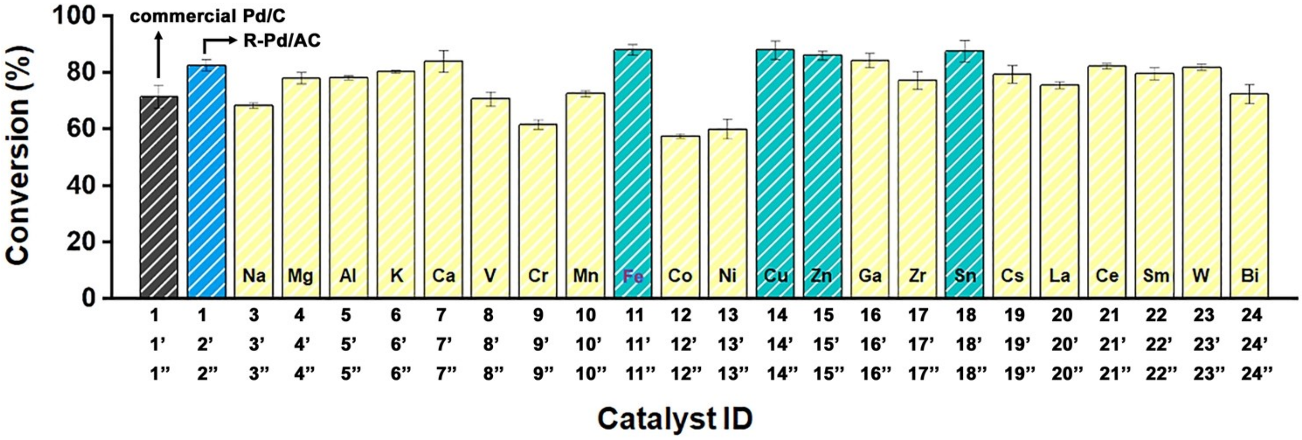

Figure S11. Conversion (%) of 24 Pd-based catalysts evaluated using the automated robotic platform. Error bars represent the standard deviation of three independent replicate experiments (n = 3) conducted for each catalyst.

This journal is © The Royal Society of Chemistry 20xx

Please do not adjust margins

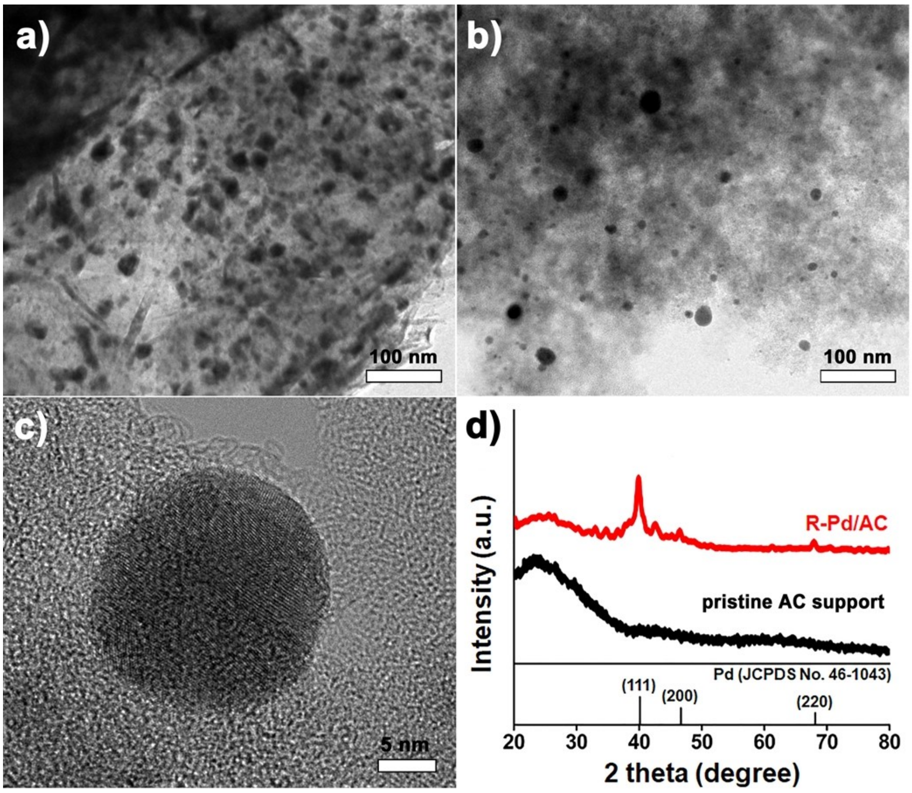

Figure S12. (a) TEM image of commercial Pd/C. (b) TEM and (c) high-resolution TEM images of the synthesized R-Pd/AC catalyst. (d) XRD patterns of R-Pd/AC compared with the pristine activated carbon support.

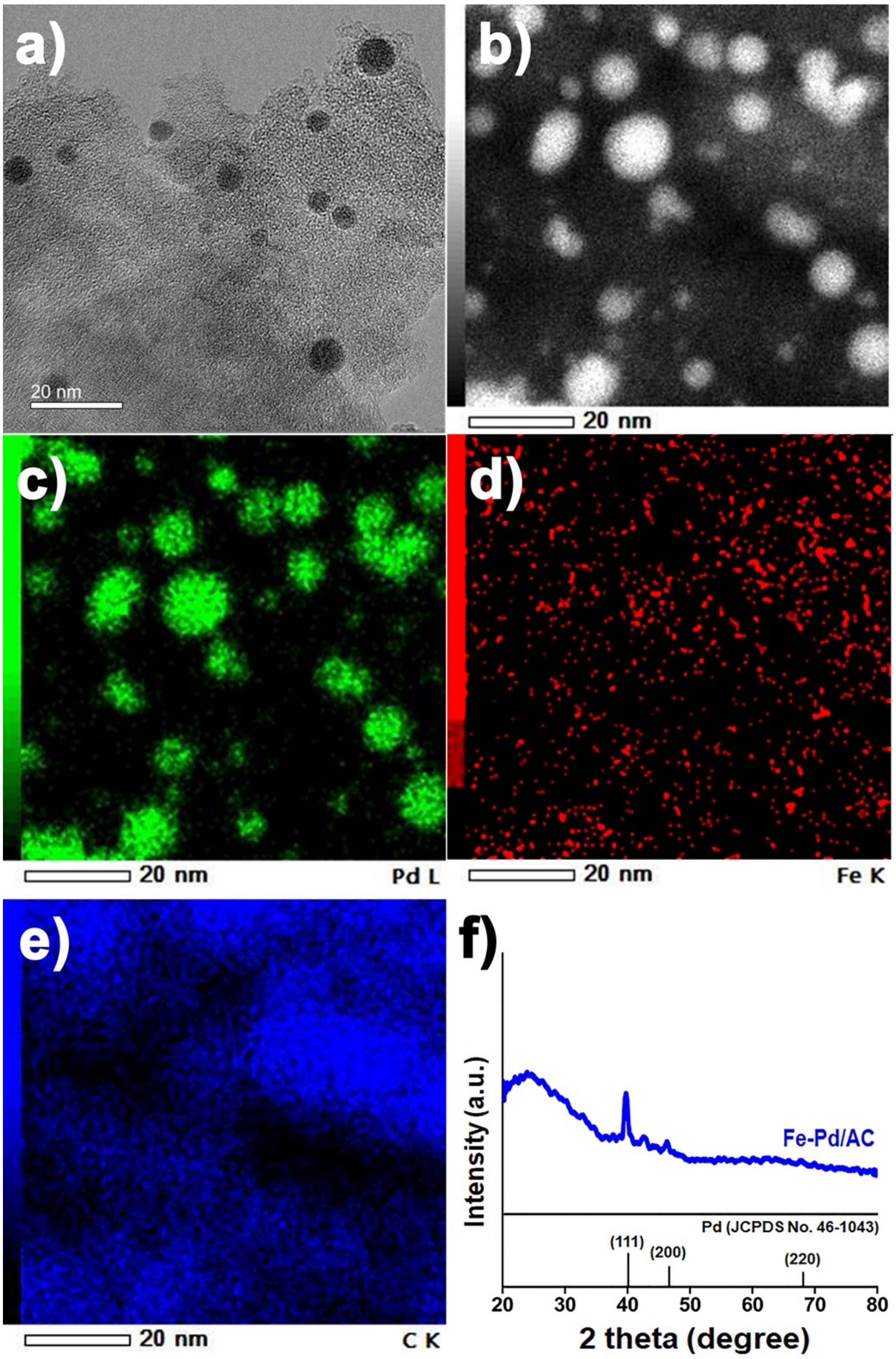

Figure S13. (a) TEM image and (b) HAADF-STEM image of Fe-Pd/AC. (c-e) Elemental mapping images: (c) Pd, (d) Fe, and (e) C. (f) XRD pattern of the Fe-Pd/AC catalyst.

This journal is © The Royal Society of Chemistry 20xx

Please do not adjust margins

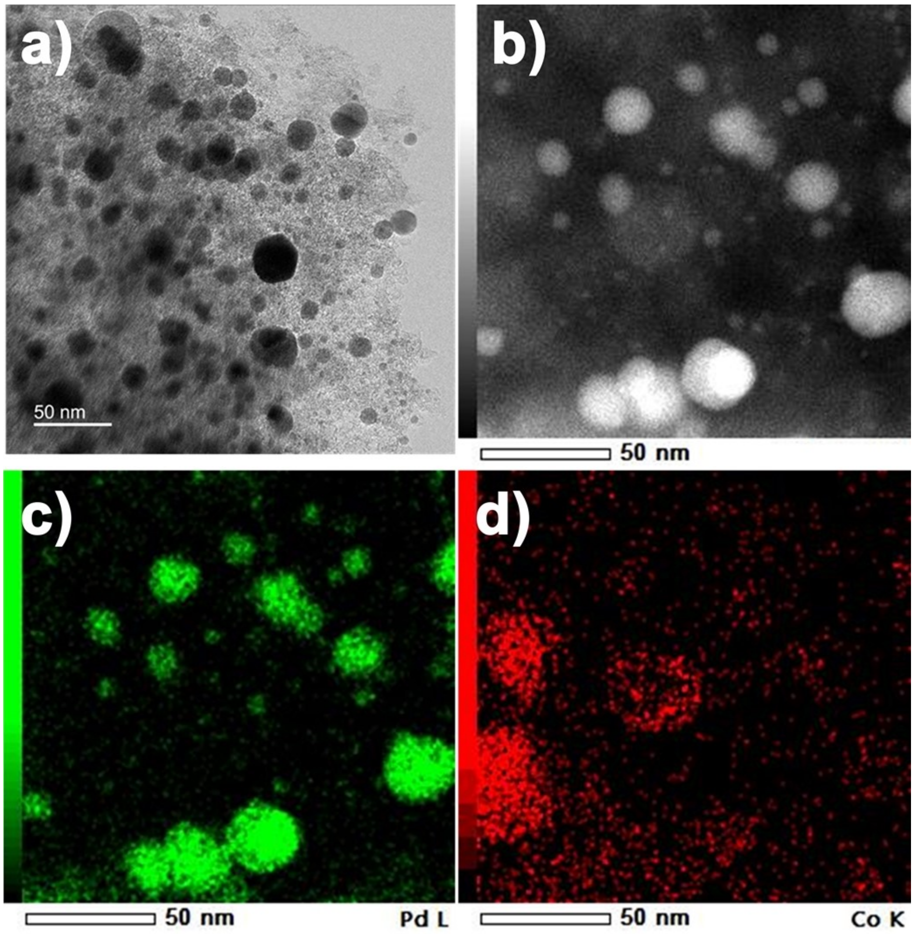

Figure S14. (a) TEM image and (b) HAADF-STEM image of Co-Pd/AC. (c-d) Elemental mapping images of the catalyst: (c) Pd and (d) Co.

Please do not adjust margins

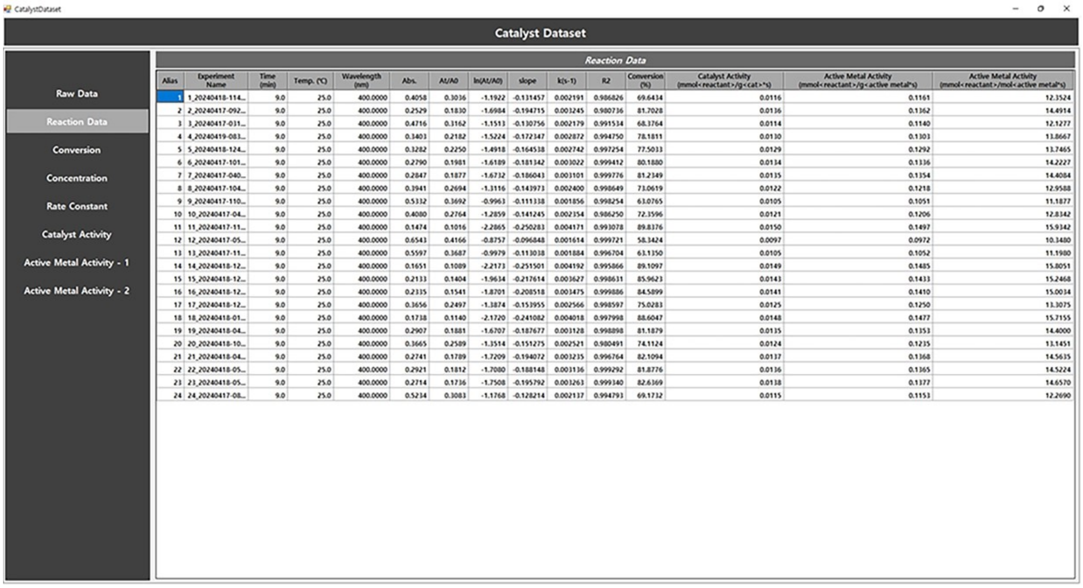

Alias	Experiment Name	Time (min)	Temp. (℃)	Wavelength (nm)	Abs.	At/A0	ln(At/A0)	slope	k(s-1)	R2	Conversion (%)	Catalyst Activity (mmol<reactant>/g<cat>*s)	Active Metal Activity (mmol<reactant>/g<active metal*s)	Active Metal Activity (mmol<reactant>/mol<active metal*s)
1	1_20240418-114...	9.0	25.0	400.0000	0.4058	0.3036	-1.1922	-0.131457	0.002191	0.986826	69.6434	0.0116	0.1161	12.3524
2	2_20240417-092...	9.0	25.0	400.0000	0.2529	0.1830	-1.6984	-0.194715	0.003245	0.980736	81.7028	0.0136	0.1362	14.4914
3	3_20240417-031...	9.0	25.0	400.0000	0.4716	0.3162	-1.1513	-0.130756	0.002179	0.991534	68.3764	0.0114	0.1140	12.1277
4	4_20240419-083...	9.0	25.0	400.0000	0.3403	0.2182	-1.5224	-0.172347	0.002872	0.994750	78.1811	0.0130	0.1303	13.8667
5	5_20240418-124...	9.0	25.0	400.0000	0.3282	0.2250	-1.4918	-0.164538	0.002742	0.997254	77.5033	0.0129	0.1292	13.7465
6	6_20240417-101...	9.0	25.0	400.0000	0.2790	0.1981	-1.6189	-0.181342	0.003022	0.999412	80.1880	0.0134	0.1336	14.2227
7	7_20240417-040...	9.0	25.0	400.0000	0.2847	0.1877	-1.6732	-0.186043	0.003101	0.999776	81.2349	0.0135	0.1354	14.4084
8	8_20240417-104...	9.0	25.0	400.0000	0.3941	0.2694	-1.3116	-0.143973	0.002400	0.998649	73.0619	0.0122	0.1218	12.9588
9	9_20240417-110...	9.0	25.0	400.0000	0.5332	0.3692	-0.9963	-0.111338	0.001856	0.998254	63.0765	0.0105	0.1051	11.1877
10	10_20240417-04...	9.0	25.0	400.0000	0.4080	0.2764	-1.2859	-0.141245	0.002354	0.986250	72.3596	0.0121	0.1206	12.8342
11	11_20240417-11...	9.0	25.0	400.0000	0.1474	0.1016	-2.2865	-0.250283	0.004171	0.993078	89.8376	0.0150	0.1497	15.9342
12	12_20240417-05...	9.0	25.0	400.0000	0.6543	0.4166	-0.8757	-0.096848	0.001614	0.999721	58.3424	0.0097	0.0972	10.3480
13	13_20240417-11...	9.0	25.0	400.0000	0.5597	0.3687	-0.9979	-0.113038	0.001884	0.996704	63.1350	0.0105	0.1052	11.1980
14	14_20240418-12...	9.0	25.0	400.0000	0.1651	0.1089	-2.2173	-0.251501	0.004192	0.995866	89.1097	0.0149	0.1485	15.8051
15	15_20240418-12...	9.0	25.0	400.0000	0.2133	0.1404	-1.9634	-0.217614	0.003627	0.998631	85.9623	0.0143	0.1433	15.2468
16	16_20240418-12...	9.0	25.0	400.0000	0.2335	0.1541	-1.8701	-0.208518	0.003475	0.999886	84.5899	0.0141	0.1410	15.0034
17	17_20240418-12...	9.0	25.0	400.0000	0.3656	0.2497	-1.3874	-0.153955	0.002566	0.998597	75.0283	0.0125	0.1250	13.3075
18	18_20240418-01...	9.0	25.0	400.0000	0.1738	0.1140	-2.1720	-0.241082	0.004018	0.997998	88.6047	0.0148	0.1477	15.7155
19	19_20240418-04...	9.0	25.0	400.0000	0.2907	0.1881	-1.6707	-0.187677	0.003128	0.998898	81.1879	0.0135	0.1353	14.4000
20	20_20240418-10...	9.0	25.0	400.0000	0.3665	0.2589	-1.3514	-0.151275	0.002521	0.980491	74.1124	0.0124	0.1235	13.1451
21	21_20240418-04...	9.0	25.0	400.0000	0.2741	0.1789	-1.7209	-0.194072	0.003235	0.996764	82.1094	0.0137	0.1368	14.5635
22	22_20240418-05...	9.0	25.0	400.0000	0.2921	0.1812	-1.7080	-0.188148	0.003136	0.999292	81.8776	0.0136	0.1365	14.5224
23	23_20240418-05...	9.0	25.0	400.0000	0.2714	0.1736	-1.7508	-0.195792	0.003263	0.999340	82.6369	0.0138	0.1377	14.6570
24	24_20240417-08...	9.0	25.0	400.0000	0.5234	0.3083	-1.1768	-0.128214	0.002137	0.994793	69.1732	0.0115	0.1153	12.2690

Figure S15. Data summary table generated by the automated UV-Vis data processing software. The dataset includes 24 catalyst experiments with key reaction metrics: absorbance (Abs.), concentration ratio (A_t/A_0), natural logarithm of the concentration ratio ($\ln(A_t/A_0)$), linear regression slope, pseudo-first-order rate constant (k), regression coefficient (R^2), and conversion efficiency (%). Calculated values for catalyst activity and active metal activity are also provided for quantitative comparison among catalyst samples.

This journal is © The Royal Society of Chemistry 20xx

Please do not adjust margins
Please do not adjust margins

Please do not adjust margins

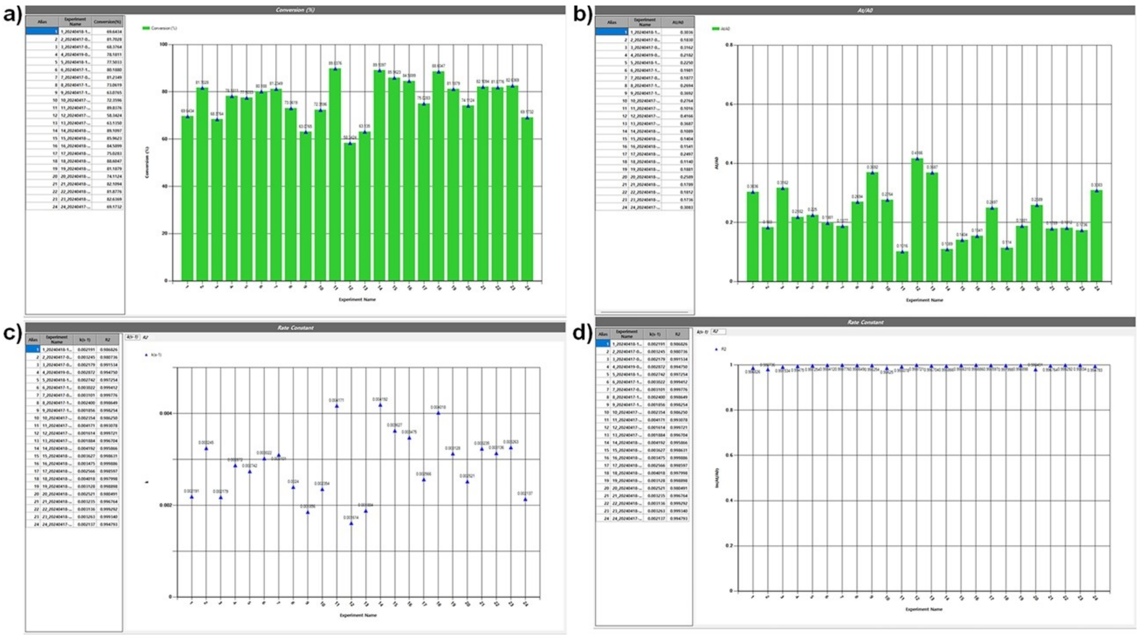

Figure S16. Real-time visualization of kinetic metrics automatically generated from the 24-sample catalyst screening. (a) Conversion (%) profile of 24 selected experiments at the 9 min mark, showing consistent catalytic performance for Fe-Pd/AC. (b) A_t/A_0 values confirming the extent of reaction progress. (c) Pseudo-first-order rate constants (k) calculated from linear fitting of $\ln(A_t/A_0)$ versus time. (d) R^2 values for kinetic fitting.

Please do not adjust margins

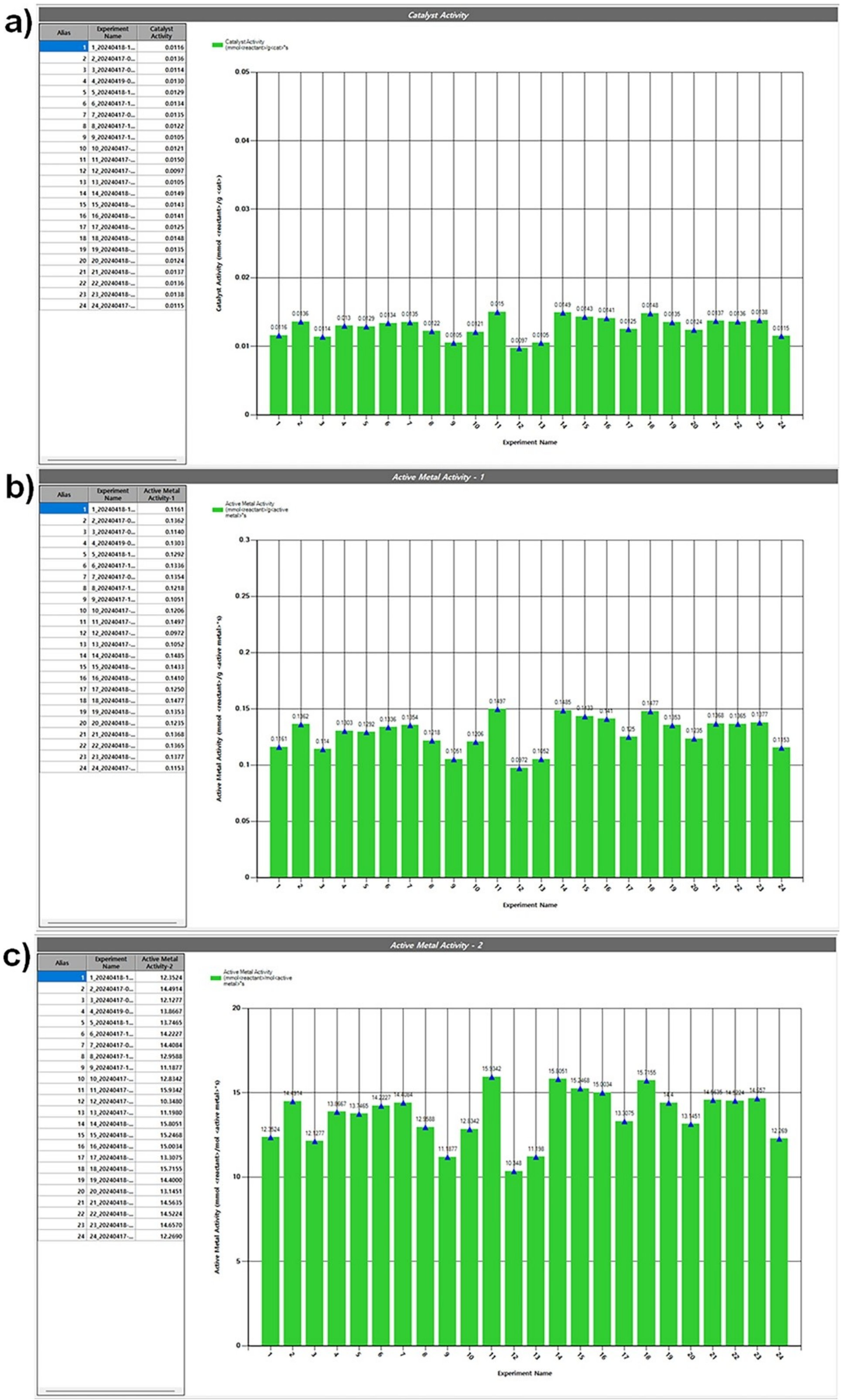

Figure S17. Quantitative analysis of catalytic activity and normalized performance metrics for 24 catalyst samples. (a) Catalyst activity calculated as mmol of reactant converted per gram of catalyst per minute. (b) Active metal activity normalized by the weight of active Pd metal (mmol·g_{Pd}^{-1}·min^{-1}). (c) Active metal activity normalized by the molar amount of Pd (mmol·mol_{Pd}^{-1}·min^{-1}).

This journal is © The Royal Society of Chemistry 20xx

Please do not adjust margins

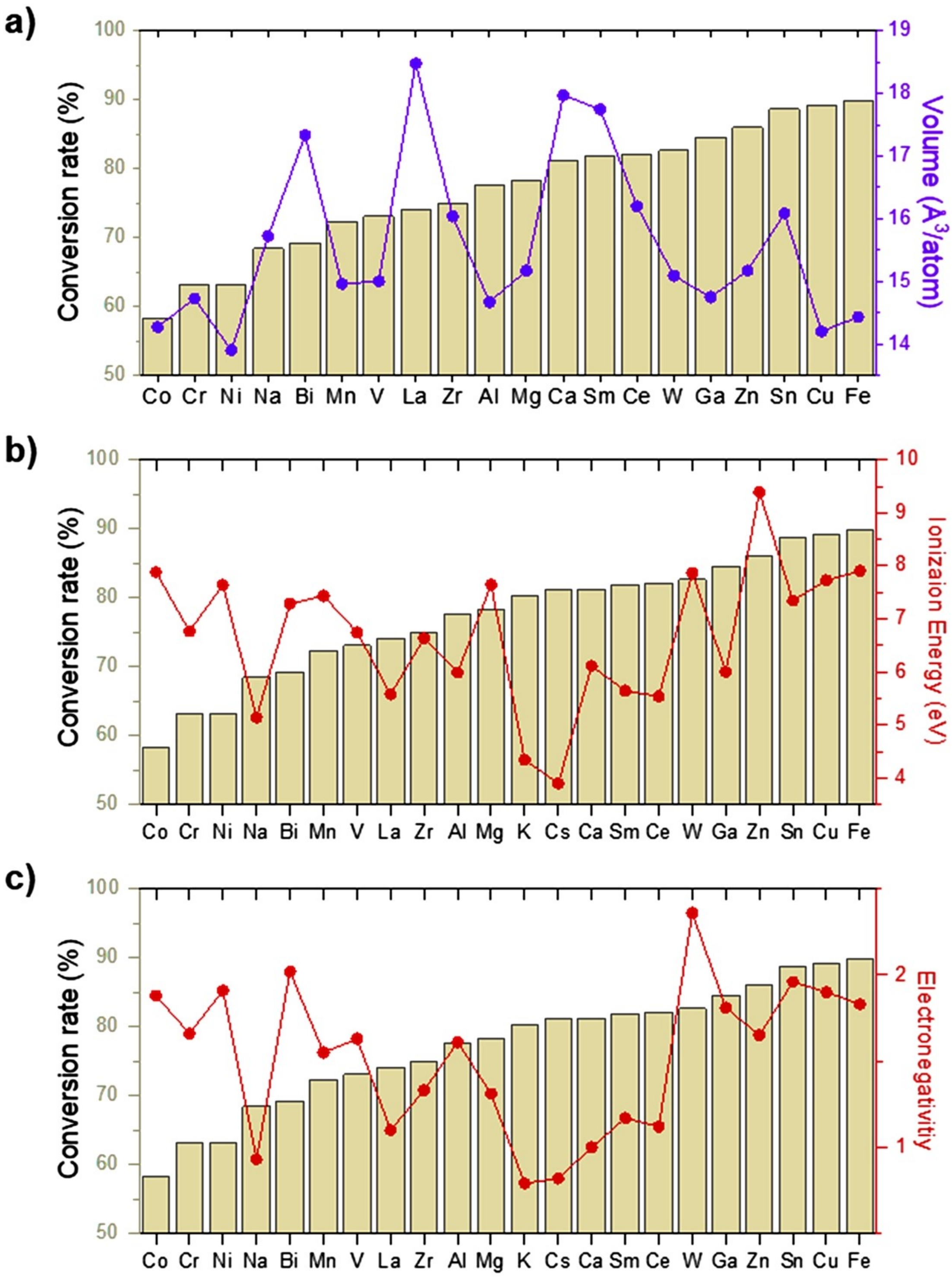

Figure S18. (a) Volume per atom of M-Pd alloys, (b) ionization energy, and (c) electronegativity (Pauling) of the M metals, plotted in order of increasing M-Pd/AC conversion rate, shown as background bars.

Please do not adjust margins

Please do not adjust margins

Table S1. Catalyst compositions and additive precursor information for 22 M-Pd/AC nanocatalysts.*

No.	Additive precursor	Chemical formula	Additive metal	Catalyst
1	Sodium hydroxide	$NaOH$	Na	Na-Pd/AC
2	Magnesium(II) nitrate hexahydrate	$Mg(NO_3)_2\cdot 6H_2O$	Mg	Mg-Pd/AC
3	Aluminum(III) nitrate nonahydrate	$Al(NO_3)_2\cdot 9H_2O$	Al	Al-Pd/AC
4	Potassium hydroxide	KOH	K	K-Pd/AC
5	Calcium chloride dihydrate	$CaCl_2\cdot 2H_2O$	Ca	Ca-Pd/AC
6	Vanadium(III) chloride	VCl_3	V	V-Pd/AC
7	Chromium(III) nitrate nonahydrate	$Cr(NO_3)_3\cdot 9H_2O$	Cr	Cr-Pd/AC
8	Manganese(II) chloride tetrahydrate	$MnCl_2\cdot 4H_2O$	Mn	Mn-Pd/AC
9	Iron(II) acetate	$Fe(CH_3COO)_2$	Fe	Fe-Pd/AC
10	Cobalt(II) acetate tetrahydrate	$Co(CH_3COO)_2\cdot 4H_2O$	Co	Co-Pd/AC
11	Nickel(II) chloride hexahydrate	$NiCl_2\cdot 6H_2O$	Ni	Ni-Pd/AC
12	Copper(II) chloride dihydrate	$CuCl_2\cdot 2H_2O$	Cu	Cu-Pd/AC
13	Zinc nitrate hexahydrate	$Zn(NO_3)_2\cdot 6H_2O$	Zn	Zn-Pd/AC
14	Gallium(III) nitrate hydrate	$Ga(NO_3)_3\cdot xH_2O$	Ga	Ga-Pd/AC
15	Zirconium(IV) chloride (anhydrous)	$ZrCl_4$	Zr	Zr-Pd/AC
16	Tin(II) chloride	$SnCl_2$	Sn	Sn-Pd/AC
17	Cesium carbonate	Cs_2CO_3	Cs	Cs-Pd/AC
18	Lanthanum(III) nitrate hexahydrate	$La(NO_3)_3\cdot 6H_2O$	La	La-Pd/AC
19	Cerium(III) nitrate hexahydrate	$Ce(NO_3)_3\cdot 6H_2O$	Ce	Ce-Pd/AC
20	Samarium(III) nitrate hexahydrate	$Sm(NO_3)_3\cdot 6H_2O$	Sm	Sm-Pd/AC
21	Tungstic acid	H_2WO_4	W	W-Pd/AC
22	Bismuth(III) nitrate pentahydrate	$Bi(NO_3)_3\cdot 5H_2O$	Bi	Bi-Pd/AC

*All metal promoters were added at a fixed 10 mol % relative to the moles of Pd in the Pd/AC catalyst. This ratio was maintained consistently across all wet-impregnation procedures to ensure uniform metal loading conditions and allow direct comparison of promoter effects.

This journal is © The Royal Society of Chemistry 20xx

Please do not adjust margins

Please do not adjust margins

Table S2. The calculated material properties of the M-Pd binary and M unary systems extracted from the Materials Project database. (E_f : formation energy, ε_d : *d*-band center, Φ : work function, I.E. : ionization energy, EN : electronegativity (Pauling)).

M	M-Pd MP-id	M Ratio	E_f (eV/atom)	Volume (Å^3/atom)	ε_d (eV)	M MP-id	Φ (eV)	I.E.	EN
Na	976971	0.250	0.000	15.723	0.907	10172	2.654	5.1391	0.93
Mg	976976	0.167	-0.332	15.169	-0.804	153	3.552	7.6462	1.31
Al	605663	0.167	-0.462	14.672	-0.508	134	4.037	5.9858	1.61
K	1180861	0.667	0.132	39.238	6.341	972981	2.108	4.3407	0.82
Ca	11826	0.167	-0.366	17.971	-0.602	45	2.774	6.1132	1
V	1216296	0.200	-0.157	15.003	0.873	146	4.110	6.7462	1.63
Cr	865786	0.250	-0.127	14.726	0.233	90	4.107	6.7665	1.66
Mn	31138	0.250	-0.229	14.959	0.340	35	4.320	7.434	1.55
Fe	21845	0.250	-0.108	14.429	-0.681	13	4.310	7.9024	1.83
Co	1183687	0.250	0.002	14.271	0.398	102	4.855	7.881	1.88
Ni	973983	0.250	0.068	13.902	-0.156	23	4.980	7.6398	1.91
Cu	1184119	0.250	-0.051	14.202	0.267	30	4.534	7.7264	1.9
Zn	1215579	0.200	-0.231	15.174	0.231	79	4.006	9.3942	1.65
Ga	1184278	0.250	-0.443	14.749	-3.019	142	4.081	5.9993	1.81
Zr	30842	0.250	-0.852	16.041	0.014	131	3.571	6.6339	1.33
Sn	718	0.250	-0.493	16.085	-2.647	117	4.072	7.3439	1.96
Cs	-	-	-	-	-	1	2.033	3.8939	0.79
La	30753	0.167	-0.597	18.479	-1.660	26	2.635	5.5769	1.1
Ce	1025415	0.125	-0.485	16.199	-0.094	28	2.977	5.5387	1.12
Sm	357	0.250	-0.854	17.747	0.031	69	2.741	5.6437	1.17
W	1219940	0.200	0.007	15.091	0.778	91	4.469	7.864	2.36
Bi	30463	0.250	-0.233	17.334	0.764	23152	4.060	7.2856	2.02

Please do not adjust margins

Table S3. Components of the robotic system, including commercial instruments and custom-designed parts, with manufacturer and model information.

Component	Description	Manufacturer/ Model	Photograph
Robotic arm	6-axis collaborative robot	Rainbow Robotics/ RB5-850	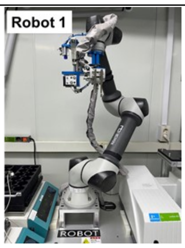 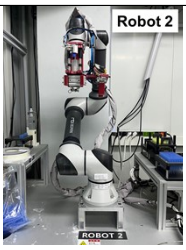
UV-Vis spectrometer	190-1100 nm spectral range with dual deuterium and tungsten-halogen light sources, capable of full-spectrum acquisition within ~20 ms using PDA technology	PerkinElmer/ Lambda 465	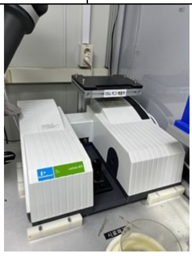
Peristaltic pump	Water transfer	Labscitech/ LEPP600F+YT15	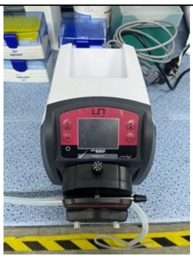
Auxiliary robot	Pump-hose manipulation and transport	Wlkata/ Mirobot	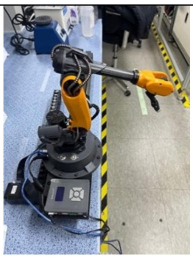
Liquid-handling robot	Automated dispensing of small-volume liquids	Opentrons/ OT-2	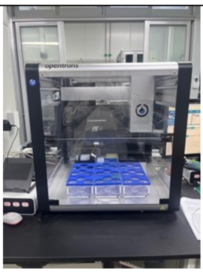
Shaker	For homogenizing reaction solutions by shaking the reaction vessels	Jeiotech/ OS-2000	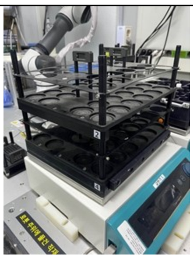

This journal is © The Royal Society of Chemistry 20xx

Please do not adjust margins

Please do not adjust margins

Cuvettes	Disposable polypropylene cuvettes for UV-Vis measurements (1 cm optical pathlength)	Kartell/ KA.1939	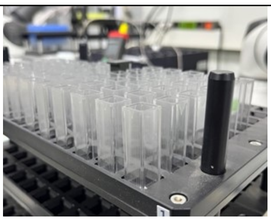
Thermal controller	Heating-block temperature control unit	HanYoungNux/ NX4-04	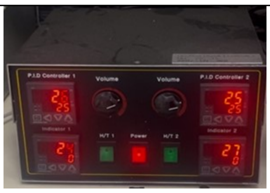
Jig components	Racks for cuvette, reaction vessels, pipette tips	Custom-designed	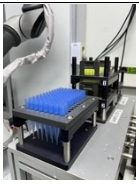 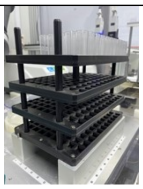 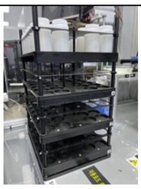
Gripper	Versatile gripper for cuvette transport, pipetting operations, and jig replacement	Custom-designed	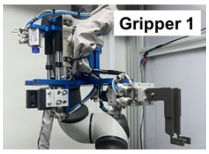 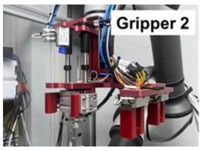
Detachable heating plate and heating block	Reaction temperature control	Custom-designed	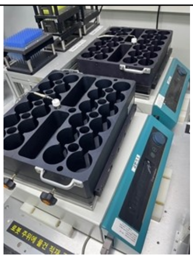 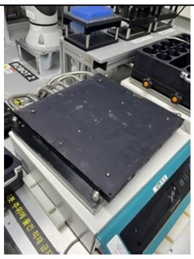

Please do not adjust margins

Please do not adjust margins

Movie S1. Conceptual 3D animation illustrating the operation of the automated robotic platform.

Movie S2. Main actions for jig handling by robot 1 and robot 2.

Movie S3. Integrated screen recording of the real-time data processing software and CCTV footage of the robotic evaluation system during unattended operation.

Movie S4. Video showing the operation of the automated pipetting device.

Movie S5. Demonstration video of the automated liquid dispensing module.

This journal is © The Royal Society of Chemistry 20xx

Please do not adjust margins
